# Student of Games: A unified learning algorithm for both perfect and imperfect information games

**DOI:** 10.1126/sciadv.adg3256

**Published:** 2023-11-15

**Authors:** Martin Schmid, Matej Moravčík, Neil Burch, Rudolf Kadlec, Josh Davidson, Kevin Waugh, Nolan Bard, Finbarr Timbers, Marc Lanctot, G. Zacharias Holland, Elnaz Davoodi, Alden Christianson, Michael Bowling

**Affiliations:** ^1^ EquiLibre Technologies, Prague, Czechia.; ^2^ Google Deepmind.; ^3^ Sony AI, New York, NY, USA.; ^4^ Amii, Edmonton, Canada.; ^5^ Midjourney, South San Francisco, CA, USA.; ^6^ Google Deepmind, Montreal, Canada.; ^7^ University of Alberta, Edmonton, Canada.

## Abstract

Games have a long history as benchmarks for progress in artificial intelligence. Approaches using search and learning produced strong performance across many perfect information games, and approaches using game-theoretic reasoning and learning demonstrated strong performance for specific imperfect information poker variants. We introduce Student of Games, a general-purpose algorithm that unifies previous approaches, combining guided search, self-play learning, and game-theoretic reasoning. Student of Games achieves strong empirical performance in large perfect and imperfect information games—an important step toward truly general algorithms for arbitrary environments. We prove that Student of Games is sound, converging to perfect play as available computation and approximation capacity increases. Student of Games reaches strong performance in chess and Go, beats the strongest openly available agent in heads-up no-limit Texas hold’em poker, and defeats the state-of-the-art agent in Scotland Yard, an imperfect information game that illustrates the value of guided search, learning, and game-theoretic reasoning.

## Teaser

Student of Games combines search, learning, and game-theoretic reasoning to play chess, go, poker, and Scotland Yard.

## INTRODUCTION

In the 1950s, Arthur L. Samuel developed a checkers-playing program that used what is now called minimax search (with alpha-beta pruning) and “rote learning” to improve its evaluation function via self-play (*1*). This investigation inspired many others, and ultimately Samuel cofounded the field of artificial intelligence (AI) (*2*) and popularized the term “machine learning.” A few years ago, the world witnessed a computer program defeat a long-standing professional at the game of Go (*3*). AlphaGo also combined learning and search. Many similar achievements happened in between, such as the race for super-human chess leading to DeepBlue (*4*) and TD-Gammon teaching itself to play master-level performance in Backgammon through self-play (*5*), continuing the tradition of using games as canonical markers of mainstream progress across the field.

Throughout the stream of successes, there is an important common element: the focus on a single game. DeepBlue could not play Go, and Samuel’s program could not play chess. Likewise, AlphaGo could not play chess; however, its successor AlphaZero (*6*) could, and did. AlphaZero demonstrated that a single algorithm could master three different perfect information games—where the game’s state is known to all players—using a simplification of AlphaGo’s approach, and with minimal human knowledge. Despite this success, AlphaZero could not play poker, and the extension to imperfect information games was unclear.

Meanwhile, approaches taken to achieve super-human poker AI were substantially different. Strong poker play has relied on game-theoretic reasoning to ensure that private information is concealed effectively. Initially, super-human poker agents were based primarily on computing approximate Nash equilibria offline (*7*). Search was then added and proved to be a crucial ingredient to achieve super-human success in no-limit variants (*8*–*10*)]. Training for other large games has also been inspired by game-theoretic reasoning and search, such as Hanabi (*11*, *12*), The Resistance (*13*), Bridge (*14*), AlphaStar (*15*), and (no-press) Diplomacy (*16*–*18*). Here again, however, despite remarkable success, each advance was still on a single game, with some clear uses of domain-specific knowledge and structure to reach strong performance.

Here, we introduce Student of Games (SoG), an algorithm that generalizes the class of games in which strong performance can be achieved using self-play learning, search, and game-theoretic reasoning. SoG uses growing-tree counterfactual regret minimization (GT-CFR): an anytime local search that builds subgames nonuniformly, expanding the tree toward the most relevant future states while iteratively refining values and policies. In addition, SoG uses sound self-play: a learning procedure that trains value-and-policy networks using both game outcomes and recursive sub-searches applied to situations that arose in previous searches.

SoG achieves strong performance in multiple challenge domains with both perfect and imperfect information—an important step toward truly general algorithms that can learn in arbitrary environments. Applications of traditional search suffer well-known problems in imperfect information games [(*2*), section 5.6.2]. Evaluation has remained focused on single domains (e.g., poker) despite recent progress toward sound search in imperfect information games (*8*, *19*, *20*). SoG fills this gap, using a single algorithm with minimal domain-specific knowledge. Its search is sound (*20*) across these fundamentally different game types: It is guaranteed to find an approximate Nash equilibrium by re-solving subgames to remain consistent during online play, and yields low exploitability in practice in small games where exploitability is computable. SoG demonstrates strong performance across four different games: two perfect information (chess and Go) and two imperfect information (poker and Scotland Yard). Finally, unlike poker, Scotland Yard has substantially longer search horizons and game lengths, requiring long-term planning.

### Background and terminology

SoG will be presented using the Factored-Observation Stochastic Games (FOSG) formalism. For further details on the formalism, see (*21*, *22*).

A game between two players starts in a specific world state *w*^init^ and proceeds to the successor world states *w* ∈ 𝒲 as a result of players choosing actions *a* ∈ 𝒜 until the game is over when a terminal state is reached. A world state can be categorized as a decision node, a terminal node, or a chance node. At a decision node, player 𝒫(*w*) acts. A terminal node marks the end of a game where no players act. A chance node is a special node representing a stochastic event, such as a die roll, with a fixed distribution. At any world state *w*, 𝒜(*w*) ⊆ 𝒜 refers to those actions that are available, or legal, in world state *w*. Sequences of actions taken along the course of the game are called histories and denoted *h* ∈ ℋ, with *h*′ ⊑ *h* denoting a prefix history (subsequence). At terminal histories, *z* ⊂ ℋ, each player *i* receives a utility *u_i_*(*z*).

An information state is a state with respect to one player’s information. Specifically, *s_i_* ∈ 𝒮*_i_* for player *i* is a set of histories that are indistinguishable due to missing information. A simple example is a specific decision point in poker where player *i* does not know the opponent’s private cards; the histories in the information state are different only in the chance event outcomes that determine the opponent’s private cards, since everything else is public knowledge. A player *i* plays a policy π*_i_* : 𝒮*_i_* → Δ(𝒜), where Δ(𝒜) denotes the set of probability distributions over actions 𝒜. The goal of each player is to find a policy that maximizes their own expected utility.

Every time a player takes an action, each player gets a private observation 𝒪_priv(*i*)_(*w*, *a*, *w*′) and a public observation 𝒪_pub_(*w*, *a*, *w*′) as a result of applying action *a*, changing the game’s state from *w* to *w*′. In perfect information games, the public observation contains complete information, i.e., 𝒪_pub_(*w*, *a*, *w*′) = (*w*, *a*, *w*′), making any private observations uninformative. Furthermore, the transition function depends only on the active player’s action, i.e., 𝒯(*w*, *a*) = 𝒯[*w*, *a*_𝒫(*w*)_]. In contrast, imperfect information games have information asymmetry between players and some players will receive informative private observations. A public state *s*_pub_(*h*) ∈ 𝒮_pub_ is the sequence of public observations encountered along the history *h*. For example, a public state in Texas hold’em poker is represented by initial public information (stack sizes and antes), the betting history, and any publicly revealed board cards. Let 𝒮*_i_*(*s*_pub_) be the set of possible information states for player *i* given *s*_pub_: Each information state *s_i_* ∈ 𝒮*_i_*(*s*_pub_) is consistent with public observations in *s*_pub_ but has different sequences of private observations. The Supplementary Materials shows a full example of a FOSG in fig. S1 and an example public tree in fig. S2.

**Fig. 1.**
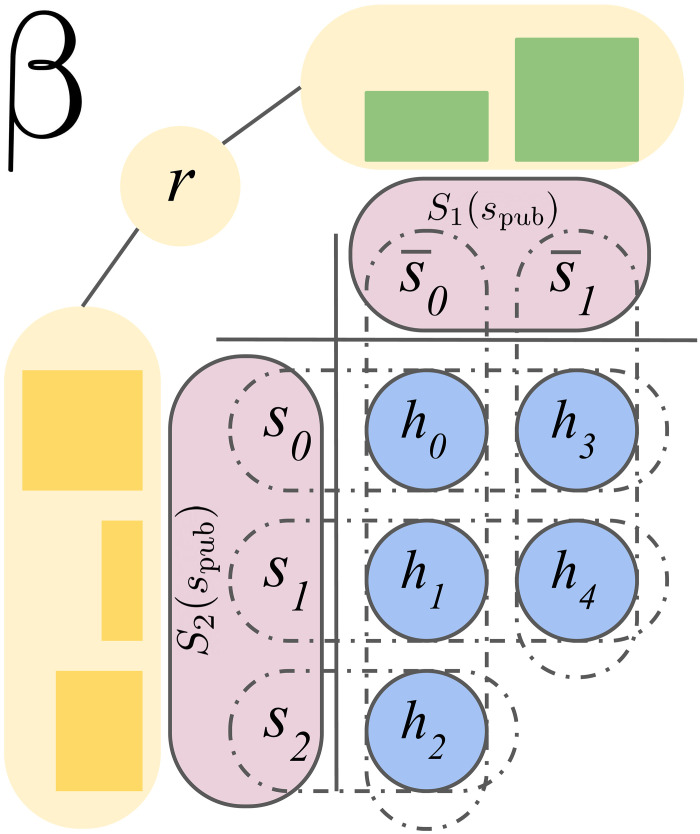
An example structure of public belief state β = (*s*_pub_, *r*). *s*_pub_ translates to two sets of information states, one for player 1, $S_{1}(s_{\mathrm{pub}})=\{{\bar{s}}_{0},{\bar{s}}_{1}\}$, and one for player 2, 𝒮_2_(*s*_pub_) = {*s*_0_, *s*_1_, *s*_2_}. Each information state includes different partitions of possible histories. Finally, *r* contains reach probabilities for information states for both players.

Imperfect information games introduce additional complexity, as 𝒮*_i_*(*s*_pub_) can now contain multiple information states that the player’s policy depends on. For example, in poker, the information states would contain the private cards of player *i*. Since past actions can leak otherwise private information, agents must reason about which information states players could be in to act soundly. A public belief state is defined as β = (*s*_pub_, *r*), where the range (or beliefs) *r* ∈ Δ[𝒮_1_(*s*_pub_)] × Δ[𝒮_2_(*s*_pub_)] is a pair of distributions over possible information states for both players representing the beliefs over information states in *s*_pub_. A basic depiction of the various components of a public belief state is depicted in Fig. 1.

Suppose players use a policy profile π = (π_1_, π_2_). Denote the expected utility to player to player *i* as *u_i_*(π_1_, π_2_) and −*i* as the opponent of player *i*. A best response to a specific opponent policy π_−*i*_ is any policy $\pi_{i}^{b}$ that achieves maximal utility against π_−*i*_: $\pi_{i}^{b}$ ∈ {π*_i_*∣*u_i_*(π*_i_*, π_−*i*_) = max_π_*i*′__*u_i_*(π_*i*′_, π_−*i*_)}. A policy profile π is a Nash equilibrium if and only if π_1_ is a best response to π_2_ and π_2_ is a best response to π_1_. There are also approximate equilibria: π is an ɛ-Nash equilibrium if and only if $u_{i}(\pi_{i}^{b},\pi_{-i})-u_{i}(\pi_{i},\pi_{-i})\leq \varepsilon$ for all players *i*.

In two-player zero-sum games, Nash equilibria are optimal because they maximize worst-case utility guarantees for both players. This worst-case utility is unique and is called the game’s value. For such games, a standard metric to represent empirical convergence rate is a strategy’s exploitability: how much, on average, a player will lose against a best response relative to a Nash equilibrium. For a given policy profile in a two-player zero-sum game π = (π_1_, π_2_), Exploitability(π) = [max_π_1′__*u*_1_(π_1′_, π_2_) + max_π_2′__*u*_2_(π_1_, π_2′_)]/2. Also, equilibrium strategies in two-player zero-sum games are interchangeable: If π*^A^* and π*^B^* are Nash equilibria, then ($\pi_{1}^{A}$, $\pi_{2}^{B}$) and ($\pi_{1}^{B}$, $\pi_{2}^{A}$) are also both equilibria. These properties mean that a Nash equilibrium plays perfect defence: It will not lose on expectation against any opponent, even one that is playing a best response to the Nash equilibrium. If the opponent makes mistakes, then the Nash equilibrium policy can win. Thus, it is reasonable for an agent to compute and play a Nash equilibrium, or an approximation to one with low exploitability.

Although the FOSG formalism generalizes beyond two-player zero-sum games, the theoretical guarantee of Nash equilibria outside of this setting is less meaningful and it is unclear how effective they would be (for example, in games with more than two players). Here, we focus on the two-player zero-sum setting. To put SoG in context, we begin with a high-level description of several techniques that have been dominant in this setting, and then contrast SoG with existing work.

### Tree search and machine learning

The first major milestones in the field of game-playing AI were obtained by efficient search techniques inspired by the minimax theorem (*1*, *4*). In a two-player zero-sum game with perfect information, the approach uses depth-limited search starting from the current world state *w_t_*, along with a heuristic evaluation function to estimate the value of states beyond the depth limit, *h*(*w*_*t* + *d*_), and game-theoretic reasoning to back up values (*23*). Researchers developed notable search enhancements (*24*, *25*) that greatly improved performance, leading to IBM’s super-human DeepBlue chess program (*4*).

This classical approach was, however, unable to achieve super-human performance in Go, which has substantially larger branching factor and state space complexity than chess. Prompted by the challenge of Go (*26*), researchers proposed Monte Carlo tree search (MCTS) (*27*, *28*). Unlike minimax search, MCTS builds trees via simulations, starting with an empty tree rooted by *w_t_* and expanding the tree by adding the first state encountered in simulated trajectories that is not currently in the tree, and finally estimating values from rollouts to the end of the game. MCTS led to substantially stronger play in Go and other games (*29*), attaining 6 dan amateur level in Go. However, heuristics leveraging domain knowledge were still necessary to achieve these milestones.

In AlphaGo (*3*), value functions and policies are incorporated, learned initially from human expert data, and then improved via self-play. A deep network approximates the value function, and a prior policy helps guide the selection of actions during the tree search. The approach was the first to achieve super-human level play in Go (*3*). AlphaGo Zero removed the initial training from human data and Go-specific features (*30*). AlphaZero reached state-of-the-art performance in chess and Shogi as well as Go, using minimal domain knowledge (*6*).

SoG, like AlphaZero, combines search and learning from self-play, using minimal domain knowledge. Unlike MCTS, which is not sound for imperfect information games, SoG’s search algorithm is based on counterfactual regret minimization and is sound for both perfect and imperfect information games.

### Game-theoretic reasoning and counterfactual regret minimization

In imperfect information games, the choice of strategies that arise from hidden information can be crucial to determining each player’s expected rewards. Simply playing too predictably can be problematic: In the classic example game of Rock, Paper, Scissors, the only thing a player does not know is the choice of the opponent’s action; however, this information fully determines their achievable reward. A player choosing to always play one action (e.g., rock) can be easily beaten by another playing the best response (e.g., paper). The Nash equilibrium plays each action with equal probability, which minimizes the benefit of any particular counter-strategy. Similarly, in poker, knowing the opponent’s cards or their strategy could yield substantially higher expected reward, and in Scotland Yard, players have a higher chance of catching the evader if their current location is known. In these examples, players can exploit any knowledge of hidden information to play the counter-strategy resulting in higher reward. Hence, to avoid being exploited, players must act in a way that does not reveal their own private information. We call this general behavior game-theoretic reasoning because it emerges as the result of computing (approximate) minimax-optimal strategies. Game-theoretic reasoning has been paramount to the success of competitive poker AI over the last 20 years.

One algorithm for computing approximate optimal strategies is counterfactual regret minimization (CFR) (*31*). CFR is a self-play algorithm that produces policy iterates $\pi_{i}^{t}$(*s*, ·) ∈ Δ(𝒜) for each player *i* at each of their information states *s* in a way that minimizes long-term average regret. As a result, the (appropriately weighted) average policy over all *T* iterations ${\bar{\pi }}^{T}$ converges to an ɛ-Nash equilibrium at a rate of $O(1/\sqrt{T})$. At each iteration, *t*, counterfactual values $v_{i}^{t}$(*s*, *a*) are computed for each action *a* ∈ 𝒜(*s*) and immediate regrets for not playing *a*, *r^t^*(*s*, *a*) = $v_{i}^{t}$(*s*, *a*) − ∑_*a*∈𝒜(*s*)_π*^t^*(*s*, *a*)$v_{i}^{t}$(*s*, *a*), are computed and tabulated in a cumulative regret table storing *R^T^*(*s*, *a*) = $\sum_{t=1}^{T}$*r^t^*(*s*, *a*). A new policy is computed using regret-matching (*32*): $\pi^{t+1}(s,\cdot )=\frac{{\left[R^{t}(s,\cdot )\right]}^{+}}{\sum_{a}{\left[R^{t}(s,a)\right]}^{+}}$, where [*x*]^+^ = max (*x*,0), if ∑*_a_*[*R^t^*(*s*, *a*)]^+^ > 0, or the uniform distribution otherwise.

CFR^+^ (*33*) is a successor of CFR that played a key role in solving the game of heads-up limit hold’em poker, the largest imperfect information game to be solved to date (*34*). A key change in CFR^+^ is a different policy update mechanism, regret-matching^+^, which defines cumulative values slightly differently: *Q^t^*(*s*, *a*) = [*Q*^*t*−1^(*s*, *a*) + *r^t^*(*s*, *a*)]^+^ and π^*t*+1^(*s*, *a*) = *Q^t^*(*s*, *a*)/∑*_b_Q^t^*(*s*, *b*).

A common form of CFR (or CFR^+^) is one that traverses the public tree of public states, rather than the classical extensive-form game tree of world states (and information states). Quantities required to compute counterfactual values, such as each player’s probabilities of reaching each information state under their policy (called their range), are maintained as beliefs. Finally, leaf nodes can be evaluated directly using the ranges, chance probabilities, and utilities [often more efficiently (*35*)].

### Imperfect information search, decomposition, and re-solving

Solution concepts like Nash equilibria and minimax are defined over policy profiles. A player’s policy is fixed during play and solely a function of the information state. Search could instead be described as a process, which might return different action distributions at subsequent visits to the same state. That is, when using search, the resulting policy can depend on more than the just the information state, such as time-limits, nondeterministic computation, stochastic events from either the game or within the search, or the outcome of other searches. These factors introduce important subtleties such as solution compatibility across different searches (*20*).

CFR has been traditionally used as a game-solving engine, computing entire policies via self-play. Each iteration traverses the entire game tree or a sampled subtree, recursively computing the counterfactual values for an information state from the values of its successor states. Suppose one wanted a policy for a part of the game up to some depth *d* > 0. If there was an oracle to compute the counterfactual values each player would receive at depth *d*, then each iteration of CFR could be run to depth *d* and query the oracle to return the values. As a result, the policies would not be available at depths *d*′ > *d*. Summarizing the policies below depth *d* by a set of values, which can be used to reconstruct policies at depth *d* and beyond, is the basis of decomposition in imperfect information games (*36*). A subgame in an imperfect information game is a game rooted at a public state *s*_pub_. In order for a subgame to be a proper game, it is paired with a belief distribution *r* over initial information states, *s* ∈ 𝒮*_i_*(*s*_pub_). This is a strict generalization of subgames in perfect information games, where every public state has exactly one information state (which is no longer private as a result) and a singleton belief with probability 1 for both players.

**Fig. 2.**
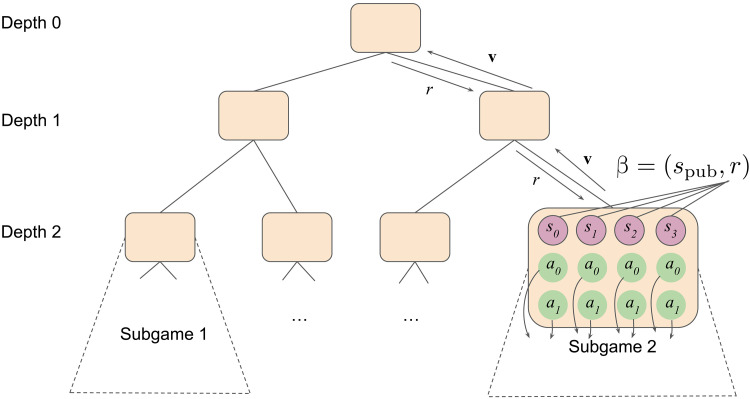
An example of depth-limited CFR solving using decomposition in a game with two specific subgames shown. Standard CFR would require traversing all the subgames. Depth-limited CFR decomposes the solve into running down to depth *d* = 2 and using **v** = **v**_**θ**_(β) to represent the second subgame’s values. On the downward pass, ranges *r* are formed from policy reach probabilities. Values are passed back up to tabulate accumulating regrets. Re-solving a subgame would require construction of an auxiliary game (*36*) (not shown).

Subgame decomposition has been a crucial component of most recent developments of poker AI that scale to large games such as no-limit Texas hold’em (*8*–*10*, *37*). Subgame decomposition enables local search to refine the policy during play analogously to the search algorithms in perfect information games and traditional Bellman-style bootstrapping to learn value functions (*8*, *13*, *38*). Specifically, a counterfactual value network (CVN) represented by parameters θ encodes the value function v_θ_(β) = {*v_i_*(*s_i_*)}_*s_i_*∈𝒮*_i_*(*s*_pub_),*i*∈{1,2}_, where β includes player’s beliefs over information states for the public information at *s*_pub_. The function v_θ_ can then be used in place of the oracles mentioned above to summarize values of the subtrees below *s*_pub_. An example of depth-limited CFR solving using decomposition is shown in Fig. 2.

Safe re-solving is a technique that generates subgame policies from only summary information of a previous (approximate) solution: a player’s range and their opponent’s counterfactual values. This is done by constructing an auxiliary game with specific constraints. The subgame policies in the auxiliary game are generated in a way that preserves the exploitability guarantees of the original solution, so they can replace the original policies in the subgame. Thorough examples of the auxiliary game construction are found in (*36*) and [(*19*), section 4.1].

Continual re-solving is an analog of classical game search, adapted to imperfect information games, that uses repeated applications of safe re-solving to play an episode of a game (*8*). It starts by solving a depth-limited game tree rooted at the beginning of the game, and search is a re-solving step. As the game progresses, for every subsequent decision at some information state *s_i_*, continual re-solving will refine the current strategy by re-solving at *s_i_*. Like other search methods, it is using additional computation to more thoroughly explore a specific situation encountered by the player.

### Related work

SoG combines many elements that were originally proposed in AlphaZero and its predecessors, as well as DeepStack (*3*, *6*, *8*, *30*). Specifically, SoG uses the combined search and learning using deep neural networks from AlphaGo and DeepStack, along with game-theoretic reasoning and search in imperfect information games from DeepStack. The use of public belief states and decomposition in imperfect information games has been a critical component of success in no-limit Texas Hold’em poker (*8*–*10*, *19*, *36*, *37*, *39*). The main difference from AlphaZero is that the search and self-play training in SoG are also sound for imperfect information games and evaluation across game types. The main difference from DeepStack is the use of substantially less domain knowledge: the use of self-play (rather than poker-specific heuristics) to generate training data and a single network for all stages of the game. The most closely related algorithm is Recurrent Belief-based Learning (ReBeL) (*37*). Like SoG, ReBeL combines search, learning, and game-theoretic reasoning via self-play. The main difference is that SoG is based on (safe) continual re-solving and sound self-play. To achieve ReBeL’s guarantees, its test-time search must be conducted with the same algorithm as in training, whereas SoG can use any belief-based value-and-policy network of the form described in Counterfactual Value-and-Policy Networks (CVPNs) (similarly to, e.g., AlphaZero, which trains using 800 simulations but then can use substantially larger simulation limits at test-time, which is needed for strong performance in many perfect information domains). SoG is also validated empirically across different challenge games of different game types, whereas ReBeL is only evaluated on two imperfect information games.

There has been considerable work in search for imperfect information games. One method that has been quite successful in practice is determinization: At decision-time, a set of candidate world states is sampled, and some form of search is performed (*40*, *41*). The baseline player we use to compare SoG to in Scotland Yard, PimBot (*42*, *43*), is based on these methods and achieved state-of-the-art results. However, these methods are not guaranteed to converge to an optimal strategy over time. We demonstrate this lack of convergence in practice over common search algorithms and standard reinforcement learning (RL) benchmarks in Supplementary Text. In contrast, the search in SoG is based on game-theoretic reasoning. Other algorithms have proposed adding game-theoretic reasoning to search: Smooth UCT (*44*) combines Upper Confidence Bounds applied to Trees (UCT) (*27*) with fictitious play; however, its convergence properties are not known. Online Outcome Sampling (*45*) derives an MCTS variant of Monte Carlo CFR (*46*); however, OOS is only guaranteed to approach an approximate equilibrium at a single information state (local consistency) and has not been evaluated in large games. GT-CFR used by SoG makes use of sound search based on decomposition and is globally consistent (*20*, *36*).

There have been a number of RL algorithms that have been proposed for two-player zero-sum games: Fictitious Self-Play (*47*), Policy-Space Response Oracles (PSRO) (*48*), Double Neural CFR (*49*), Deep CFR and DREAM (*50*, *51*), Regret Policy Gradients (*52*), Exploitability Descent (*53*), Neural Replicator Dynamics (NeuRD) (*54*), Advantage Regret-Matching Actor Critic (*55*), Friction FoReL (*56*), Extensive-form Double Oracle (XDO) (*57*), Neural Auto-curricula (NAC) (*58*), and Regularized Nash Dynamics (R-NaD) (*59*). These methods adapt classical algorithms for computing (approximate) Nash equilibria to the RL setting with sampled experience and general function approximation. As such, they combine game-theoretic reasoning and learning. Several of these methods have shown promise to scale: Pipeline PSRO defeated the best openly available agent in Stratego Barrage; ARMAC showed promising results on large poker games. R-NaD truly demonstrated scale by obtaining human-level performance in the very large game of Stratego (*59*). In Starcraft, AlphaStar was able to use human data and game-theoretic reasoning to create a master-level real-time strategy policy (*15*). However, none of them can use search at test-time to refine their policy; this shifts a learning and function approximation burden onto training, typically making these methods more computationally demanding in both training time and model capacity to encode a policy or value function.

Last, there have been works that use some combination of search, learning, and/or game-theoretic reasoning applied to specific domains. Neural networks have been trained via Q-learning to learn to play Scotland Yard (*60*); however, the overall play strength of the resulting policy was not directly compared to any other known Scotland Yard agent. In poker, Supremus proposed a number of improvements to DeepStack and demonstrated that they make a big difference when playing human experts (*38*). Another work used a method inspired by DeepStack applied to The Resistance (*13*). In the cooperative setting, several works have made use of belief-based learning (and search) using public subgame decomposition (*12*, *61*, *62*), applied to Hanabi (*11*). Learning and game-theoretic reasoning were also recently combined to produce agents that play well with humans without human data on the collaborative game Overcooked (*63*). Search and RL were combined to produce a bridge bidding player that cooperated with a state-of-the-art bot (WBridge5) and with humans (*14*). Of considerable note is the game of (no-press) Diplomacy. In that game, game-theoretic reasoning was combined with learning in Best Response Policy Iteration (*16*), and game-theoretic search and supervised learning were combined in (*17*) reaching human-level performance on the two-player variant. Recently, all three were combined in DORA (*18*), which learned to play Diplomacy without human data reaching human-level performance on the two-player variant, and subsequently, Cicero (*64*) reached human-level performance on the full game including communication with humans via language models. The main difference between SoG and these works is that they focus on specific games and exploit domain-specific knowledge to attain strong performance.

### Descriptions of challenge domains

Chess and Go are well-known classic games, both seen as grand challenges of AI (*4*, *26*) that have driven progress in AI since its inception. The achievement of DeepBlue beating Kasparov in 1997 is widely regarded to be the first big milestone of AI. Today, chess playing computer programs remain consistently super-human, and one of the strongest and most widely used programs is Stockfish (*65*). Go emerged as the favorite new challenge domain, which was particularly difficult for classical search techniques (*26*). MCTS (*27*–*29*) emerged as the dominant search technique in Go. The best of these programs, Crazy Stone and Zen, were able to reach the level of 6 dan amateur (*3*). It was not until 2016 that AlphaGo defeated the first human professional Lee Sedol in the historical 2016 match, and also defeated the top human Ke Jie in 2017.

Heads-up no-limit Texas hold’em is the most common two-player version of poker played by humans, which is also played by DeepStack and Libratus (*8*, *9*). Human expert-level poker has been the standard challenge domain among imperfect information games, inspiring the field of game theory itself. No-limit Texas hold’em presents the complexity of stochastic events (card draws), imperfect information (private cards), and a very large state space (*66*). Here, we use blinds of 100 and 50 chips, and stack sizes of 200 big blinds (20,000 chips).

Scotland Yard is a compelling board game of imperfect information, receiving a Spiel des Jahres award in 1983 as well as being named the “The most popular game ’83” by SpielBox (*67*). The game is played on a map of London, where locations are connected by edges representing different modes of transportation. One player plays as “Mr. X” (the evader) and others control detectives (pursuers). Mr. X is only visible on specific rounds, but detectives get to see the mode of transportation Mr. X uses every round (e.g., taxi, bus, and subway). To win, detectives need to catch Mr. X within 24 rounds. Scotland Yard is a perfect example of an imperfect information game that requires search for strong play—the detectives have to plan multiple moves into the future while reasoning about possible locations that Mr. X may be. Similarly, although Mr. X has perfect information, he must also reason about where he could be to, for example, avoid revealing his location. Unlike poker, Scotland Yard has partially observable actions, so private information is effected by the agents’ choices in addition to chance.

This suite of games covers the classic challenge domains across game types (perfect information and imperfect information, some with stochastic elements and others not), as well as an additional challenging imperfect information game with substantially longer sequences of actions and a fundamentally different type of uncertainty over hidden actions.

## RESULTS

To understand the results, we give a brief high-level overview of our main algorithm, SoG, which we present formally in Materials and methods.

### SoG: Algorithm summary

The SoG algorithm trains the agent via sound self-play: Each player, when faced with a decision to make, uses a sound GT-CFR search equipped with a CVPN to generate a policy for the current state, which is then used to sample an action to take.

GT-CFR grows a tree, starting with the current public state, and consists of two alternating phases: The regret update phase runs public tree CFR updates on the current tree; the expansion phase expands the tree by adding new public states via simulation-based expansion trajectories. One iteration of GT-CFR consists of one run of the regret update phase followed by one run of the expansion phase.

The self-play process generates two types of training data for updating the value and policy networks: search queries, which are public belief states that were queried by the CVPN during the GT-CFR regret update phase, and full-game trajectories from the self-play games. The search queries must be solved to compute counterfactual value targets for updating the value network. The full-game trajectories provide targets for updating the policy network. In practice, the self-play data generation and training happen in parallel: Actors generate the self-play data (and solve queries), while trainers learn new networks and periodically update the actors.

### Theoretical results

We have two main theoretical results, which we describe here only informally. They are formally treated in Materials and Methods. Theorem 1 ensures that the exploitability of the final GT-CFR policy is at most $O(1/\sqrt{T})$, where *T* is the number of GT-CFR iterations, under some conditions on how the search tree is expanded and so long as the value function is reasonably accurate. SoG invokes GT-CFR to re-solve a subtree every time it must act. Theorem 2 bounds the exploitability of the entire SoG policy, proving that it is sound to use GT-CFR recursively. Both theorems together ensure that the search is sound up to some acceptable error in the value function. If there is no error in the value function, and the values are the game-theoretic optimal values, then GT-CFR will probably converge to a Nash equilibrium strategy if run under the conditions stated in the theorems.

### Experimental results

We evaluate SoG on four games: chess, Go, heads-up no-limit Texas hold’em poker, and Scotland Yard. We also evaluate SoG on the commonly used small benchmark poker game Leduc hold’em, and a custom-made small Scotland Yard map, where the approximation quality compared to the optimal policy can be computed exactly.

When reporting the results, we use the notation SoG(*s*, *c*) for SoG running GT-CFR with *s* total expansion simulations, and *c* expansion simulations per regret update phase, so the total number of GT-CFR iterations is then $\frac{s}{\left\lceil c\right\rceil }$. For example, SoG(8000,10) refers to 8000 expansion simulations at 10 expansions per regret update (800 GT-CFR iterations). We choose this notation style to be easily comparable to number of simulations in AlphaZero.

### Exploitability in Leduc poker and small Scotland Yard map

Supporting our theoretical results, we empirically evaluate the exploitability of SoG in Leduc poker (*68*) and in Scotland Yard on a small map named “glasses.” The full description of Leduc poker is presented in Supplementary Text, and the map is illustrated in fig. S3.

Exploitability is a function of a specific (fixed) policy profile. However, for a search algorithm like SoG, previous searches may affect policies computed at later points within the same game, as explained in the “Imperfect information search, decomposition, and re-solving” section. Hence, we construct multiple samples of the SoG policy by choosing a random seed, running the search algorithm at every public state in a breadth-first manner such that every search is conditioned on previous searches at predecessor states, and composing together the policies obtained from each search. We then show the minimum, average, and maximum exploitabilities over policies constructed in this way from 50 different choices of seeds. If the minimum and maximum exploitability values are tight, then they represent an accurate estimate of true exploitability.

**Fig. 3.**
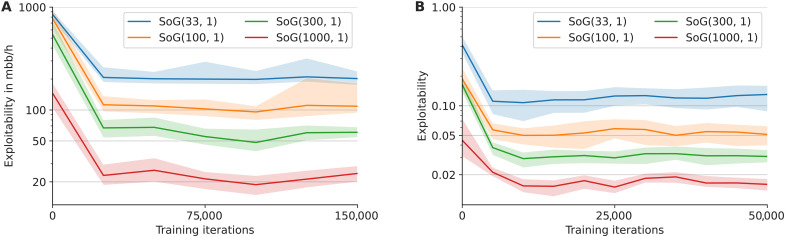
Exploitability of SoG as a function of the number of training steps under different number of simulations of GT-CFR. For both (**A**) Leduc poker and (**B**) Scotland Yard (glasses map), each line corresponds to a different evaluation condition, e.g., SoG(*s*, *c*) used at evaluation time. The ribbon shows minimum and maximum exploitability out of 50 seeded runs for each setup. The units of the *y* axis in Leduc poker are milli–big blinds per hand (mbb/h), which corresponds to one thousandth of a chip in Leduc. In Scotland Yard, the reward is either −1 (loss) or +1 (win). All networks were trained using a single training run of SoG(100,1), and the *x* values correspond to a network trained for the corresponding number of steps.

Figure 3 shows the exploitability of SoG in Leduc poker and the glasses map of Scotland Yard, as a function of the number of CVPN training steps. For these graphs, we evaluate multiple networks (each trained for a different number of steps) generated by a single training run of SoG(100,1). Each data point corresponds to a specific network (determined by number of steps trained) being evaluated under different settings during play. For each specific *x* value, a single network was used to obtain each exploitability value of SoG using the network under different evaluation conditions.

We observe that exploitability drops fairly quickly as the training steps increase. Also, even using only one CFR update per simulation, there is significant difference in exploitability when more simulations are used. As Theorem 1 suggests, more training (by reducing ɛ) and more search (by increasing *T*) reduces the exploitability of SoG. Standard RL algorithms in self-play are not guaranteed to reduce exploitability with continued training in this setting. We show this lack of convergence in practice in Supplementary Text.

### Results in challenge domains

Our main results compare the performance of SoG to other agents in our challenge domains. We trained a version of AlphaZero using its original settings in chess and Go, e.g., using 800 MCTS simulations during training, with 3500 concurrent actors each on a single TPUv4, for a total of 800,000 training steps. SoG was trained using a similar amount of TPU resources.

In chess, we evaluated SoG against Stockfish 8 level 20 (*65*) and AlphaZero. SoG(400,1) was run in training for 3 million training steps. During evaluation, Stockfish uses various search controls: number of threads and time per search. We evaluate AlphaZero and SoG up to 60,000 simulations. A tournament between all of the agents was played at 200 games per pair of agents (100 games as white, 100 games as black). From this tournament, we rank players according to their Elo ratings. Elo is a classic system for rating chess players originally designed by Arpad Elo in 1967 and still widely used today (*69*) in many games. A rating, *r_i_*, is assigned to each player *i* such that a logistic model predicts the probability of player *i* beating player *j* as 1/[1 + 10^(*r_j_*−*r_i_*)/400^]. Table 1 shows the relative Elo comparison obtained by this tournament, where a baseline of 0 is chosen for Stockfish (threads = 1, time = 0.1 s).

**Table 1.** Relative Elo of different agents in chess (left) and Go (right). Each agent played 200 matches (100 as white and 100 as black) against every other agent in the tournament. For chess, Elo of Stockfish with a single thread and 100-ms thinking time was set to be 0. For Go, Elo of GnuGo was set to be 0. The other values are relative to those. AlphaZero(*s* = 16,000, *t* = 800,000) refers to 16,000 search simulations. For full results, see tables S3 and S4.

Chess agents	Relative Elo	Go agents	Relative Elo
AlphaZero(sims = 60,000)	+592	AlphaZero(*s* = 16,000, *t* = 800,000)	+3139
Stockfish(threads = 16, time = 4 s)	+530	AlphaZero(*s* = 8,000, *t* = 800,000)	+2875
AlphaZero(sims = 8,000)	+455	SoG(*s* = 16,000, *c* = 10)	+1970
SoG(*s* = 60,000, *c* = 10)	+420	SoG(*s* = 8,000, *c* = 10)	+1902
Stockfish(threads = 4, time = 1 s)	+382	Pachi(*s* = 100,000)	+869
SoG(*s* = 8,000, *c* = 10)	+268	Pachi(*s* = 10,000)	+231
Stockfish(threads = 1, time = 0.1 s)	0	GnuGo(l = 10)	0

**Fig. 4.**
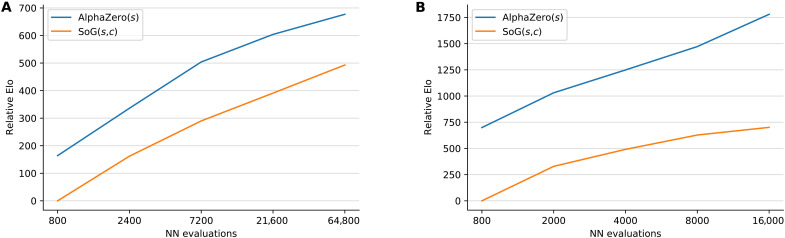
Scalability of SoG with increasing number of neural network evaluations compared to AlphaZero measured on relative Elo scale. The *x* axis corresponds to the number of simulations in AlphaZero and *s* in SoG(*s*, *c*). Elo of SoG(*s* = 800, *c*) was set to be 0. In chess (**A**), *c* = 10 for all runs, with varying *s* ∈ {800,2400,7200,21600,64800}. In Go (**B**), we graph SoG using (*s*, *c*) ∈ {(800,1), (2000,10), (4000,10), (8000,10), (16000,16).}

**Fig. 5.**
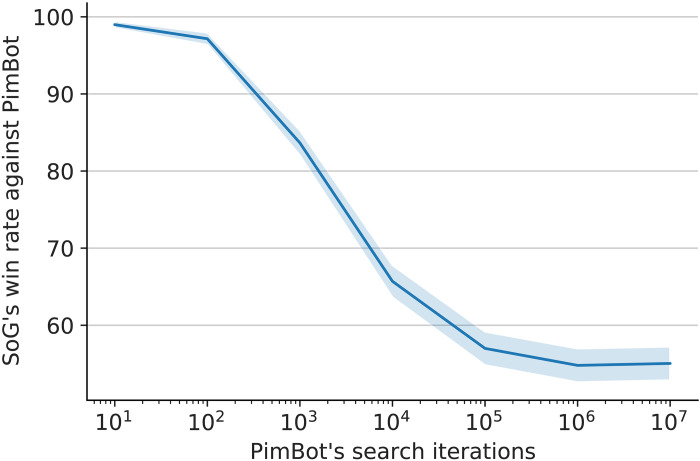
Win rate of SoG(400,1) against PimBot with varying simulations. Two thousand matches were played for each data point, with roles swapped for half of the matches. Note that the *x* axis has logarithmic scale. The ribbon shows 95% confidence interval.

**Fig. 6.**
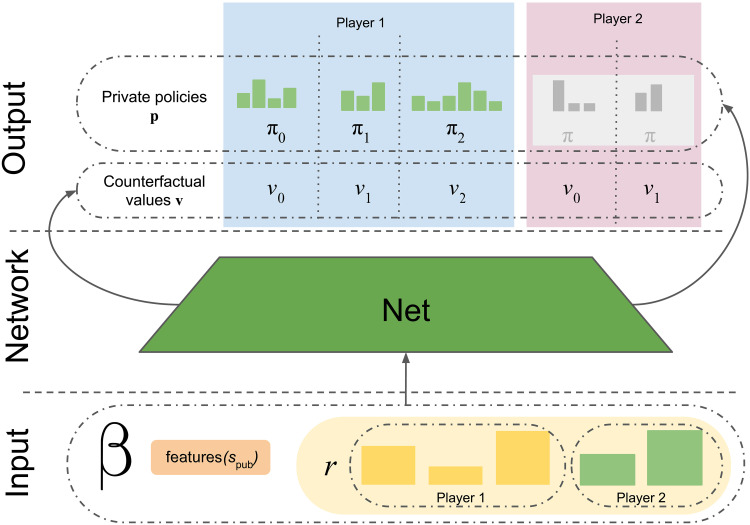
A counterfactual value-and-policy network (CVPN). Each query, β, to the network includes beliefs *r* and an encoding of *s*_pub_ to get the counterfactual values **v** for both players and policies **p** for the acting player in each information state *s_i_* ∈ *s*_pub_(*h*), producing outputs *f*_**θ**_. Since players may have different actions spaces (as in, e.g., Scotland Yard), there are two sets of policy outputs: one for each player, and **p** refers to the one for the acting player at *s*_pub_ only (depicted as player 1 in this diagram by graying out player 2’s policy output).

**Fig. 7.**
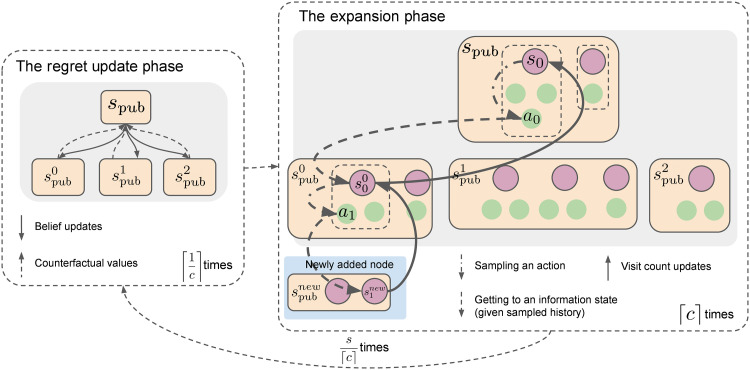
Overview of the phases in one iteration of GT-CFR. The regret update phase propagates beliefs down the tree, obtains counterfactual values from the CVPN at leaf nodes (or from the environment at terminals), and passes back counterfactual values to apply the CFR update. The expansion phase simulates a trajectory from the root to a leaf, adding public states to the tree. In this case, the trajectory starts in the public belief state *s*_pub_ by sampling the information state *s*_0_. After that, the sampled action *a*_0_ leads to the information state $s_{0}^{0}$ in public state $s_{\mathrm{pub}}^{0}$, and finally, the action *a*_1_ leads to a new public state that is added to the tree.

**Fig. 8.**
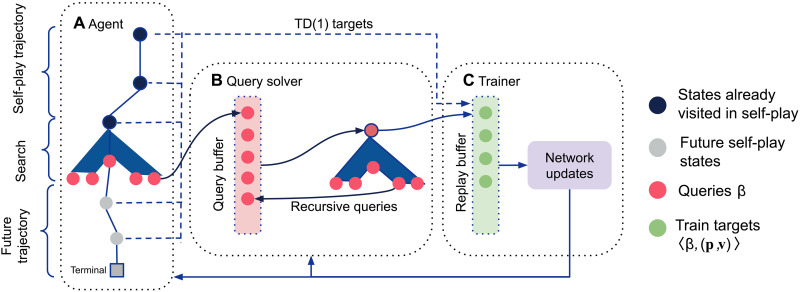
SoG training process. Actors collect data via sound self-play and trainers run separately over a distributed network. (**A**) Each search produces a number of CVPN queries with input β. (**B**) Queries are added to a query buffer and subsequently solved by a solver that studies the situation more closely via another invocation of GT-CFR. During solving, new recursive queries might be added back to the query buffer; separately, the network is (**C**) trained on minibatches sampled from the replay buffer to predict values and policy targets computed by the solver.

In Go, we evaluate SoG(60000,10) using a similar tournament as in chess, against two previous Go programs: GnuGo (at its highest level, 10) (*70*) and Pachi v7.0.0 (*71*) with 10,000 and 100,000 simulations, as well as AlphaZero (*6*) with a range of search simulations at different points in training. SoG(400,1) was used in training for 1 million training steps. Table 1 shows the relative Elo comparison for a subset of the agents that played in this tournament, where a baseline of 0 is chosen for GnuGo. The full results are presented in tables S3 and S4.

Notice in both chess and Go that SoG reaches strong performance. In chess, SoG(60000,10) is stronger than Stockfish using four threads and 1 s of search time. In Go, SoG(16000,10) is more than 1100 Elo stronger than Pachi with 100,000 simulations. Also, SoG(16000,10) wins 0.5% (2 of 400) of its games against AlphaZero(*s* = 8000, *t* = 800,000). As a result, SoG appears to be performing at the level of top human amateur, possibly even professional level. In both cases, SoG is weaker than AlphaZero, with the gap being smaller in chess. We hypothesize that this difference is the result of MCTS being more efficient than CFR on perfect information games, as the price of SoG’s generality.

For chess and Go, we also present direct Elo comparisons from a tournament between AlphaZero (trained for 800,000 steps) and SoG agents when increasing the number of neural network evaluations in Fig. 4. These results demonstrate that SoG is able to scale, improving performance with available computation. Note that while the neural network evaluations account for most of the run time, the complexity of the regret update phase is linear in the size of the tree. The run time is thus quadratic in the number of GT-CFR iterations. The absolute time cost could be reduced by an implementation that runs the regret update and expansion phase in parallel. For a more detailed analysis of SoG’s complexity, see the “Performance guarantees for continual re-solving” section. Intuitively, we would expect *c* = 1 (corresponding to one regret update per expansion simulation) to be best choice. Because of these computational constraints, we chose by hand a small number of values for *c* > 1. We did notice that *c* = 1 is not always the best choice in practice and hope to explore this more thoroughly in the future.

In heads-up no-limit Texas hold’em, we evaluate SoG against Slumbot2019 (*72*, *73*), the best open-source heads-up no-limit computer poker player. When training poker, SoG uses randomized betting abstractions described in Supplementary Text to reduce the number of actions from 20,000 to 4 or 5. SoG(10,0.01) is trained for up to 1.1 million training steps and then evaluated. Since poker has particularly high variance, we use the Action-Informed Value Assessment Tool (AIVAT) (*74*) to compute a more accurate estimate of performance. We also evaluate SoG against a local best-response (LBR) player that can use only fold and call actions with a poker-specific heuristic, which has shown to find exploits in previous poker agents (*75*). Table 2 summarizes the results of SoG along with other recent poker agents. SoG(10,0.01) wins on average 7 ± 3 milli–big blinds (0.7 chips) per hand, with 95% confidence intervals (3.1 million matches). LBR fails to find an exploit of SoG’s strategy, and SoG wins on average by 434 ± 9 milli–big blinds per hand.

**Table 2.** Head-to-head results showing expected winnings (mbb/h) of SoG and other recently published agents against Slumbot and LBR. The LBR agent use either fold or call (FC) actions in the all four rounds. The ± shows one standard error. LBR results for Slumbot are from (*75*). The other results are from the papers describing the agents.

Agent name	Slumbot	LBR (*75*)
Slumbot (2016)	–	−522 ± 50
ARMAC (*55*)	–	−460 ± 260
DeepStack (*8*)	–	428 ± 87
Modicum (*39*)	11 ± 5	–
ReBeL (*37*)	45 ± 5	–
Supremus (*38*)	176 ± 44	951 ± 96
SoG(10,0.01)	7 ± 3	434 ± 9

In Scotland Yard, the current state-of-the-art agent in this game is based on MCTS with game-specific heuristic enhancements (*42*). We call this agent “PimBot” based on its main author, Joseph Antonius Maria (“Pim”) Nijssen. PimBot implements a variant of MCTS that uses determinization, heuristic evaluations, and playout policies (*42*, *43*). PimBot won 34 of 50 manually played games against the Nintendo DS Scotland Yard AI.

In our experiment, SoG is trained up to 17 million steps. In evaluation, we play a head-to-head match with SoG(400,1) against PimBot at different number of simulations per search. The results are shown in Fig. 5. These results show that SoG is winning significantly even against PimBot with 10 million search simulations (55% win rate), compared to SoG searching a tiny fraction of the game. PimBot does not seem to play stronger with more search at this point, as both the 1 million and 10 million iteration versions have the same performance against SoG.

As in chess and Go, SoG also demonstrates strong performance in these complex imperfect information games. In the case of poker, in addition to beating Slumbot, it also beats the LBR agent, which was not possible for some previous agents (including Slumbot). Finally, SoG significantly beats the state-of-the-art agent in Scotland Yard, an imperfect information game with longer episodes and fundamentally different kind of imperfect information than in poker. Together, these results indicate that SoG is capable of strong performance across four games, two fundamentally different game types, and can act as a truly unified algorithm combining search, learning, and game-theoretic reasoning for competitive games.

## DISCUSSION

SoG is a unified algorithm that combines search, learning, and game-theoretic reasoning. SoG is composed of two main components: a GT-CFR technique and sound self-play, which learns CVPNs via self-play. Most notably, SoG is a sound algorithm for both perfect and imperfect information games: As computational resources increase, SoG is guaranteed to produce better approximation of minimax-optimal strategies. This finding is also verified empirically in Leduc poker, where additional search leads to test-time approximation refinement, unlike any pure RL algorithms that do not use search.

In addition to being sound, SoG also demonstrates strong performance on challenge domains, using minimal domain knowledge. In the perfect information games of chess and Go, SoG performs at the level of human experts or professionals, but can be substantially weaker in head-to-head play than specialized algorithms for this class of games, like AlphaZero, when given the same resources. In the imperfect information game no-limit Texas hold’em poker, SoG beats Slumbot, the best openly available poker agent, and is shown not to be exploited by an LBR agent using poker-specific heuristics. In Scotland Yard, SoG defeats the state-of-the-art agent.

There are some limitations of SoG that are worth investigating in future work. First, the use of betting abstractions in poker could be removed in favor of a general action-reduction policy for large action spaces. Second, SoG currently requires enumerating the information states per public state, which can be prohibitively expensive in some games; this might be approximated by a generative model that samples world states and operates on the sampled subset. Finally, substantial computational resources are used to attain strong play in challenge domains; an interesting question is whether this level of play is achievable with less computational resources.

## MATERIALS AND METHODS

We now give a detailed description of the SoG algorithm. As SoG has several components, we describe them each individually first, and then describe how they are all combined toward the end of the section. For clarity, many of the details (including full pseudocode) are presented in Supplementary Text.

### Counterfactual value-and-policy networks

The first major component of SoG is a CVPN with parameters **θ**, depicted in Fig. 6. These parameters represent a function *f*_**θ**_(β)** = **(**v**, **p**), where outputs **v** are counterfactual values (one per information state per player), and prior policies **p**, one per information state for the acting player, in the public state *s*_pub_(*h*) at some history of play *h*.

In our experiments, we use standard feed-forward networks and residual networks. The details of the architecture are described in Supplementary Text.

### Search via GT-CFR

GT-CFR is an algorithm that runs a CFR variant on a public game tree that is incrementally grown over time. GT-CFR starts with an initial tree, ℒ^0^, containing β and all of its child public states. Then, each iteration, *t*, of GT-CFR consists of two phases:

1) The regret update phase runs several public tree CFR updates on the current tree ℒ*^t^*.

2) The expansion phase expands ℒ*^t^* by adding new public states via simulation-based expansion trajectories, producing a new larger tree ℒ^*t*+1^.

When reporting the results, we use the notation SoG(*s*, *c*) for SoG running GT-CFR with *s* total expansion simulations, and *c* expansion simulations per regret update phase, so the total number of GT-CFR iterations is then $\frac{s}{\left\lceil c\right\rceil }$. *c* can be fractional, so, e.g., 0.1 indicates a new node every 10 regret update phases. Figure 7 depicts the whole GT-CFR cycle. We chose this specific notation to directly compare total expansion simulations, *s*, to AlphaZero.

The regret update phase runs $\left\lceil \frac{1}{c}\right\rceil$ updates (iterations) of public tree CFR on ℒ*^t^* using simultaneous updates, regret-matching ^+^, and linearly weighted policy averaging (*33*). At public tree leaf nodes, a query is made to the CVPN at belief state β′, whose values *f*_**θ**_(β′) = (**v**, **p**) are used as estimates of counterfactual values for the public subgame rooted at β′.

In the expansion phase, new public tree nodes are added to ℒ. Search statistics, initially empty, are maintained over information states *s_i_*, accumulated over all expansion phases within the same search. At the start of each simulation, an information state *s_i_* is sampled from the beliefs in β_root_. Then, a world state *w*_root_ is sampled from *s_i_*, with associated history *h*_root_. Actions are selected according to a mixed policy that takes into account learned values {via π_PUCT_[*s_i_*(*h*)]} as well as the currently active policy {π_CFR_[*s_i_*(*h*)]} from search: $\pi_{\mathrm{select}}[s_{i}(h)]=\frac{1}{2}\pi_{\mathrm{PUCT}}[s_{i}(h)]+\frac{1}{2}\pi_{\mathrm{CFR}}[s_{i}(h)]$. The first policy is determined by PUCT (*3*) using counterfactual values *v_i_*(*s_i_*, *a*) normalized by the sum of the opponent’s reach probability at *s_i_* to resemble state-conditional action values, and the prior policy **p** obtained from the queries. The second is simply CFR’s average policy at *s_i_*(*h*). As soon as the simulation encounters an information state *s_i_* ∈ *s*_pub_ such that $s_{\mathrm{pub}}\notin L$, the simulation ends, *s*_pub_ is added to ℒ, and visit counts are updated along nodes visited during the trajectory. Similarly to AlphaZero (*6*), virtual losses (*76*) are added to the PUCT statistics when doing ⌈*c*⌉ simulations inside one GT-CFR iteration.

AlphaZero always expands a single action/node at the end of the iteration (the action with the highest UCB score). Optimal policies in perfect information games can be deterministic, and expanding a single action/node is a good way to avoid unneeded computation after unpromising actions. MCTS methods are sound as long as the best action has been added, which is always true in the limit as the tree is completely filled out. In imperfect information games, optimal policies might be stochastic, having nonzero probability over multiple actions. Rather than expanding a single action, SoG thus expands the top *k* actions as ranked by the prior. We use *k* = 1 for perfect information games, where computation cost is very important and we only need to find a single good action, and *k* = ∞ to add all children for imperfect information games where it is important to mix over multiple actions. In addition to being sound in the limit, SoG also has a finite-time guarantee on policy quality when *k* = ∞.

### Modified continual re-solving

The continual re-solving method used by DeepStack (*8*) takes advantage of a few poker properties, which are not found in other games like Scotland Yard, so we use a more general re-solving method that can be applied to a broader class of games. Recall that the re-solving step and the corresponding auxiliary game requires (i) the current player’s range and (ii) the opponent’s counterfactual values. This provides a succinct and sufficient representation to safely re-solve the subgame rooted in a public state *s*_pub_. In hold’em poker, players generally take turns making actions, and a depth-limited search tree for a re-solving auxiliary game can always be deep enough to contain a state for the opponent’s action. All player actions are fully visible to both players, so the opponent’s maximum counterfactual value in the previous search tree can be used for the next re-solving auxiliary game, no matter what opponent action we are responding to. By working within the single, fixed domain of poker, these properties let DeepStack and Libratus simply retrieve the re-solving summary information from its previous search.

As SoG is a general algorithm, it can no longer leverage this special case. The current public state *s*_pub_ might not have been included in the previous search tree, so the prior computation might not directly provide us with the required summary information for re-solving the subgame rooted in *s*_pub_. SoG thus starts its re-solving process in the state closest to the current state that is included in the previous search tree: $s_{\mathrm{pub}}^{\mathrm{prev}}$. We initialize the search tree with a single branch leading from $s_{\mathrm{pub}}^{\mathrm{prev}}$ to *s*_pub_, with all off-branch actions being leaves. The search tree is then expanded forward from *s*_pub_, as in DeepStack or Libratus. This re-solving auxiliary game uses summary information for $s_{\mathrm{pub}}^{\mathrm{prev}}$ instead of *s*_pub_, and by construction, these values and probabilities are available in the previous search tree.

When generating a policy for this next re-solving auxiliary game with GT-CFR, we constrain the expansion phase of GT-CFR to only grow the tree under *s*_pub_, to focus the computation on the states relevant for the current decision. After re-solving, our action probabilities for our current information state will still come from the new re-solved policy at *s*_pub_, which might not be at the root of the search tree.

Finally, like DeepStack, the gadget for the re-solving auxiliary game is modified by mixing in the opponent’s range from the previous search. As introduced in (*36*), the gadget used to transform a subgame into a re-solving auxiliary game is a binary opponent decision for each opponent information state before the subgame. At each information state, the opponent can either terminate (T) and receive the opponent counterfactual values in the re-solving summary, or follow (F) this line of play into the corresponding subgame. The effect of the gadget is to generate an opponent range *r* for the subgame. Given an opponent range *r*^prev^ from the previous search, Moravcik *et al*. (*8*) modified the opponent range to be α*r* + (1 − α)*r*^prev^. As with DeepStack, this regularization toward the previous opponent policy empirically improves the performance, and we used α = 0.5.

### Performance guarantees for continual re-solving

Growing the tree in GT-CFR allows the search to selectively focus on parts of the space that are important for local decisions. Starting with a small tree and adding nodes over time does not have an additional cost in terms of convergence:

**Theorem 1.** Let ℒ*^t^* be the public tree at time *t*. Assume public states are never removed from the search tree, so ℒ*^t^* ⊆ ℒ^*t*+1^. For any given tree ℒ, let 𝒩(ℒ) be the interior of the tree: all nonleaf, nonterminal public states where GT-CFR generates a policy. Let ℱ(ℒ) be the frontier of ℒ, containing the nonterminal leaves where GT-CFR uses ɛ-noisy estimates of counterfactual values. Let *U* be the maximum difference in counterfactual value between any two strategies, at any information state, and *A* be the maximum number of actions at any information state. Then, the regret at iteration *T* for player *i* is bounded:

$$R_{i}^{T,\mathrm{full}}\leq \sum_{t=1}^{T}|F(L^{t})|\varepsilon +\sum_{s\;\mathrm{pub}\in N(L^{T})}|S_{i}(s\;\mathrm{pub})|U\sqrt{AT}$$

The regret *R*^*T*,full^ in Theorem 1 is the gap in performance between GT-CFR iterations and the highest-value strategy. Theorem 1 shows that the average policy returned by GT-CFR converges toward a Nash equilibrium at a rate of $1/\sqrt{T}$, but with some minimum exploitability due to ɛ-error in the value function. There is also no additional cost when using GT-CFR as the game-solving algorithm for each re-solving search step in continual re-solving:

**Theorem 2.** Assume we have played a game using continual re-solving, with one initial solve and D re-solving steps. Each solving or re-solving step finds an approximate Nash equilibrium through *T* iterations of GT-CFR using an ɛ-noisy value function, public states are never removed from the search tree, the maximum interior size ∑_*s*pub∈𝒩(ℒ*^T^*)_∣𝒮*_i_*(*s*_pub_)∣ of the tree is always bounded by *N*, the frontier size of the tree is always bounded by *F*, the maximum number of actions at any information states is *A*, and the maximum difference in values between any two strategies is *U*. The exploitability of the final strategy is then bounded by $\left(5D+2)(F\varepsilon +NU\sqrt{\frac{A}{T}}\right)$.

Theorem 2 is similar to Theorem 1 of (*8*), adapted to GT-CFR and using a more detailed error model, which can more accurately describe value functions trained on approximate equilibrium strategies. It shows that continual re-solving with GT-CFR has the general properties we might desire: Exploitability decreases with more computation time and decreasing value function error, and only increases linearly with game length. Proofs of these theorems are presented as Supplementary Text.

The computational complexity of a GT-CFR re-solving step with *T* iterations expanding *k* children is 𝒪(*kT*^2^) public states visited and CVPN network calls. In the special case of perfect information games, the number of network calls can be reduced to 𝒪(*T*).

At every iteration *t*, in the expansion phase, GT-CFR will traverse a single trajectory through the tree to expand a leaf, and use the CVPN to evaluate the newly expanded children. This requires *k* network calls and a worst case of ∣*ℒ^t^*∣ states visited or ⌈log*_b_*∣*ℒ^t^*∣⌉ states visited in a balanced *b*-ary tree.

In the regret update phase, GT-CFR visits every state in *ℒ^t^* and uses the CVPN to evaluate every leaf of the tree. Because *k* child states are added to the tree at each iteration, ∣*ℒ^t^*∣ ≤ 𝒪(*kt*), giving the stated bounds.

In perfect information games, *k* = 1, each player range is a single number, and we only need to evaluate a state once because the optimal policy does not depend on the player ranges. If a state is evaluated once with a range of 1 for both players and then stored, any other belief state can by evaluated by scaling the stored result by the opponent’s ranges.

### Data generation via sound self-play

SoG generates episodes of data in self-play by running searches at each decision point. Each episode starts at the initial history *h*_0_ corresponding to the start of the game and produces a sequence of histories (*h*_0_, *h*_1_, ⋯). At time *t*, the agent runs a local search and then selects an action *a_t_*, and the next history *h*_*t*+1_ is obtained from the environment by taking action *a_t_* at *h_t_*. Data for training the CVPN are collected via resulting trajectories and the individual searches.

When generating data for training the CVPN, it is important that searches performed at different public states be consistent with both the CVPN represented by **θ** and with searches made at previous public states along the same trajectory (e.g., two searches should not be computing parts of two different optimal policies). This is a critical requirement for sound search (*8*, *20*, *36*), and we refer to the process of a sound search algorithm generating data in self-play as sound self-play. To achieve sound self-play, searches performed during data generation run GT-CFR on the modified safe re-solving auxiliary game (as described in the “Modified continual re-solving” section).

### Training process

The quality of the policies produced by GT-CFR and data generated by sound self-play depends critically on the values returned by the CVPN. Hence, it is important for the estimates to be accurate to produce high-performance searches and generate high-quality data. In this subsection, we describe the procedure we use to train the CVPN. The process is summarized in Fig. 8.

### Query collection

As described in the “Search via GT-CFR” and “Data generation via sound self-play” sections, episodes are generated by each player running searches of GT-CFR from the current public state. Each search produces a number of network queries from public tree leaf nodes β (depicted as pink nodes in Fig. 8).

The training process improves the CVPN via supervised learning. Values are trained using Huber loss (*77*) based on value targets, and the policy loss is cross entropy with respect to a target policy. Value and policy targets are added to a sliding window dataset of training data that is used to train the CVPN concurrently. The CVPN is updated asynchronously on the actors during training.

### Computing training targets

Policy targets are assembled from the searches started at public states along the main line of episodes (the histories reached in self-play) generated by sound self-play described in the “Data generation via sound self-play” section. Specifically, they are the output policies for all information states within the root public state, computed in the regret update phase of GT-CFR.

Value targets are obtained in two different ways. First, the outcome of the game is used as a [TD(1)] value target for states along the main line of episodes generated by sound self-play. Second, value targets are also obtained by bootstrapping: running an instance of GT-CFR from subgames rooted at input queries. In principle, any solver could be used because any subgame rooted at β has well-defined values. Thus, this step acts much like a policy improvement operator via decomposition described in the “Imperfect information search, decomposition, and re-solving” section. Specifically, the value targets are the final counterfactual values after *T* iterations of GT-CFR for all the information states within the public state that initiated the search. The specific way that the different value targets are assigned is described by the pseudocode in Supplementary Text and determined by a hyperparameter noted in table S2.

### Recursive queries

While the solver is computing targets for a query, it is also generating more queries itself by running GT-CFR. Some of these recursive queries are also added to the buffer for future solving so that the CVPN can produce reasonable answers for all leaves in a search, not just those on the self-play lines. As a result, at any given time, the buffer may include queries generated by search in the main self-play game or by solver-generated queries off the main line. To ensure that the buffer is not dominated by recursive queries, we set the probability of adding a new recursive query to less than 1 (in our experiments, the value is typically 0.1 or 0.2; see table S2 for the exact values).

### Consistency of training process

One natural question is whether, or under what circumstances, the training process could ensure convergence to the optimal values? The answer is positive: The training process converges to the optimal values, asymptotically, as *T* → ∞ and with very large (exponential) memory.

Informally, imagine an oracle function *f*(β) that can simply memorize the values and policy for the particular β similar to a tabular value or policy iteration algorithm except with continuous keys. For any subgame rooted at some β with a depth of 1 (every action leads to terminal states), the values and policies can be computed and stored for β after *T* iterations of the solver. This can then be applied inductively: Since CFR is deterministic, for any subgame on the first iteration of GT-CFR, a finite number of queries will be generated. Each of these queries will be solved using GT-CFR. Eventually, the query will be a specific one that is one step from the terminal state whose values can be computed exactly and stored in *f*(β). As this value was generated in self-play or by a query solver, and CFR is deterministic, it will produce another self-play game with the identical query, except it will load the solved value from *f*(β), and inductively the values will get propagated from the bottom up. Since CFR is deterministic and *T* is finite, these ensure that the memory requirement is not infinite despite the continuous-valued keys. Practically, the success of the training process will depend on the representational capacity and training efficacy of the function approximation (i.e., neural network architecture).

For a fully detailed description of the algorithm, including hyperparameter values and specific descriptions of each process described above, see Supplementary Text.

## Supplementary Materials

### This PDF file includes:

Supplementary Text Figs. S1 to S4 Tables S1 to S6 References

## References

[1] A. L. Samuel, Some studies in machine learning using the game of checkers. IBM J. Res. Dev. 44, 206–226 (2000).

[2] S. J. Russell, P. Norvig, *Artificial Intelligence: A Modern Approach* (Pearson Education, ed. 3, 2010).

[3] D. Silver, A. Huang, C. J. Maddison, A. Guez, L. Sifre, G. van den Driessche, J. Schrittwieser, I. Antonoglou, V. Panneershelvam, M. Lanctot, S. Dieleman, D. Grewe, J. Nham, N. Kalchbrenner, I. Sutskever, T. Lillicrap, M. Leach, K. Kavukcuoglu, T. Graepel, D. Hassabis, Mastering the game of Go with deep neural networks and tree search. Nature 529, 484–489 (2016). 26819042 10.1038/nature16961

[4] M. Campbell, A. J. Hoane, F.-H. Hsu, Deep Blue. Artif. Intell. 134, 57–83 (2002).

[5] G. Tesauro, TD-Gammon, a self-teaching backgammon program, achieves master-level play. Neural Comput. 6, 215–219 (1994).

[6] D. Silver, T. Hubert, J. Schrittwieser, I. Antonoglou, M. Lai, A. Guez, M. Lanctot, L. Sifre, D. Kumaran, T. Graepel, T. Lillicrap, K. Simonyan, D. Hassabis, A general reinforcement learning algorithm that masters chess, shogi, and Go through self-play. Science 362, 1140–1144 (2018). 30523106 10.1126/science.aar6404

[7] M. B. Johanson, “Robust strategies and counter-strategies: From superhuman to optimal play,” thesis, University of Alberta, Edmonton, Alberta, Canada (2016).

[8] M. Moravcik, M. Schmid, N. Burch, V. Lisý, D. Morrill, N. Bard, T. Davis, K. Waugh, M. Johanson, M. Bowling, Deepstack: Expert-level artificial intelligence in heads-up no-limit poker. Science 358, 508–513 (2017). 10.1126/science.aam6960 28254783

[9] N. Brown, T. Sandholm, Superhuman AI for heads-up no-limit poker: Libratus beats top professionals. Science 360, 418–424 (2017). 10.1126/science.aao1733 29249696

[10] N. Brown, T. Sandholm, Superhuman AI for multiplayer poker. Science 365, 885–890 (2019). 31296650 10.1126/science.aay2400

[11] N. Bard, J. N. Foerster, S. Chandar, N. Burch, M. Lanctot, H. F. Song, E. Parisotto, V. Dumoulin, S. Moitra, E. Hughes, I. Dunning, S. Mourad, H. Larochelle, M. G. Bellemare, M. Bowling, The Hanabi challenge: A new frontier for AI research. Artif. Intell. 280, 103216 (2020).

[12] A. Lerer, H. Hu, J. Foerster, N. Brown, Improving policies via search in cooperative partially observable games. *Proceedings of the Thirty-Fourth AAAI Conference on Artificial Intelligence* (AAAI, 2020).

[13] J. Serrino, M. Kleiman-Weiner, D. C. Parkes, J. B. Tenenbaum, Finding friend and foe in multiagent games. *Proceedings of the Thirty-third Conference on Neural Information Processing Systems* (NeurIPS, 2019).

[14] E. Lockhart, N. Burch, N. Bard, S. Borgeaud, T. Eccles, L. Smaira, R. Smith, Human-agent cooperation in bridge bidding. *Proceedings of the Cooperative AI Workshop at 34th Conference on Neural Information Processing Systems* (NeurIPS, 2020).

[15] O. Vinyals, I. Babuschkin, W. M. Czarnecki, M. Mathieu, A. Dudzik, J. Chung, D. H. Choi, R. Powell, T. Ewalds, P. Georgiev, J. Oh, D. Horgan, M. Kroiss, I. Danihelka, A. Huang, L. Sifre, T. Cai, J. P. Agapiou, M. Jaderberg, A. S. Vezhnevets, R. Leblond, T. Pohlen, V. Dalibard, D. Budden, Y. Sulsky, J. Molloy, T. L. Paine, C. Gulcehre, Z. Wang, T. Pfaff, Y. Wu, R. Ring, D. Yogatama, D. Wünsch, K. McKinney, O. Smith, T. Schaul, T. Lillicrap, K. Kavukcuoglu, D. Hassabis, C. Apps, D. Silver, Grandmaster level in StarCraft II using multi-agent reinforcement learning. Nature 575, 350–354 (2019). 31666705 10.1038/s41586-019-1724-z

[16] T. W. Anthony, T. Eccles, A. Tacchetti, J. Kramár, I. M. Gemp, T. C. Hudson, N. Porcel, M. Lanctot, J. Pérolat, R. Everett, S. Singh, T. Graepel, Y. Bachrach, Learning to play no-press Diplomacy with best response policy iteration. *Thirty-third Conference on Neural Information Processing Systems* (NeurIPS, 2020).

[17] J. Gray, A. Lerer, A. Bakhtin, N. Brown, Human-level performance in no-press Diplomacy via equilibrium search. *Proceedings of the International Conference on Learning Representations* (ICLR, 2020).

[18] A. Bakhtin, D. Wu, A. Lerer, N. Brown, No-press Diplomacy from scratch. *Proceedings of the Thirty-fourth Conference on Neural Information Processing Systems* (NeurIPS, 2021).

[19] N. Brown, T. Sandholm, Safe and nested subgame solving for imperfect-information games. *Proceedings of the 31st Conference on Neural Information Processing Systems* (NIPS, 2017).

[20] M. Šustr, M. Schmid, M. Moravčík, N. Burch, M. Lanctot, M. Bowling, Sound search in imperfect information games. *Proceedings of the International Conference on Autonomous Agents and Multiagent Systems* (AAMAS, 2020).

[21] V. Kovařík, M. Schmid, N. Burch, M. Bowling, V. Lisý, Rethinking formal models of partially observable multiagent decision making. Artif. Intell. 303, 103645 (2022).

[22] M. Schmid, “Search in imperfect information games,” thesis, Charles University, Prague, Czech Republic (2021).

[23] D. E. Knuth, R. W. Moore, An analysis of alpha-beta pruning. Artif. Intell. 6, 293–326 (1975).

[24] T. A. Marsland, M. Campbell, A survey of enhancements to the alpha-beta algorithm. *ACM Annual Conference* (Association for Computing Machinery, 1981), pp. 109–114.

[25] J. Schaeffer, A. Plaat, New advances in alpha-beta searching. *ACM Conference on Computer Science* (1996), pp. 124–130.

[26] S. Gelly, L. Kocsis, M. Schoenauer, M. Sebag, D. Silver, C. Szepesvári, O. Teytaud, The grand challenge of computer Go: Monte Carlo tree search and extensions. Commun. ACM 55, 106–113 (2012).

[27] L. Kocsis, C. Szepesvári, Bandit based Monte-Carlo planning, in *Machine Learning: ECML 2006. ECML 2006. Lecture Notes in Computer Science, Vol. 4212*. J. Fürnkranz, T. Scheffer, M. Spiliopoulou, Eds. (Springer, 2006), pp. 282–293.

[28] R. Coulom, Efficient selectivity and backup operators in Monte-Carlo tree search, in *Computers and Games. CG 2006. Lecture Notes in Computer Science, Vol. 4630*. H. J. van den Herik, P. Ciancarini, H. H. L. M. J. Donkers, Eds. (Springer, 2007), pp. 72–83.

[29] C. B. Browne, E. Powley, D. Whitehouse, S. M. Lucas, P. I. Cowling, P. Rohlfshagen, S. Tavener, D. Perez, S. Samothrakis, S. Colton, A survey of monte carlo tree search methods. IEEE Trans. Comput. Intell. AI Games 4, 1–43 (2012).

[30] D. Silver, J. Schrittwieser, K. Simonyan, I. Antonoglou, A. Huang, A. Guez, T. Hubert, L. Baker, M. Lai, A. Bolton, Y. Chen, T. Lillicrap, F. Hui, L. Sifre, G. van den Driessche, T. Graepel, D. Hassabis, Mastering the game of Go without human knowledge. Nature 550, 354–359 (2017). 29052630 10.1038/nature24270

[31] M. Zinkevich, M. Johanson, M. Bowling, C. Piccione, Regret minimization in games with incomplete information. *Advances in Neural Information Processing Systems 20* (NIPS, 2008), pp. 905–912.

[32] S. Hart, A. Mas-Colell, A simple adaptive procedure leading to correlated equilibrium. Econometrica 68, 1127–1150 (2000).

[33] O. Tammelin, N. Burch, M. Johanson, M. Bowling, Solving heads-up limit Texas Hold’em. *Proceedings of the 24th International Joint Conference on Artificial Intelligence* (IJCAI, 2015).

[34] M. Bowling, N. Burch, M. Johanson, O. Tammelin, Heads-up limit Hold’em poker is solved. Science 347, 145–149 (2015). 25574016 10.1126/science.1259433

[35] M. Johanson, N. Bard, M. Lanctot, R. Gibson, M. Bowling, Efficient nash equilibrium approximation through Monte Carlo counterfactual regret minimization. *Proceedings of the Eleventh International Conference on Autonomous Agents and Multi-Agent Systems* (AAMAS, 2012).

[36] N. Burch, M. Johanson, M. Bowling, Solving imperfect information games using decomposition. *Proceedings of the Twenty-Eighth AAAI Conference on Artificial Intelligence* (AAAI, 2014).

[37] N. Brown, A. Bakhtin, A. Lerer, Q. Gong, Combining deep reinforcement learning and search for imperfect-information games. *Thirty-fourth Annual Conference on Neural Information Processing Systems* (NeurIPS, 2020).

[38] R. Zarick, B. Pellegrino, N. Brown, C. Banister, Unlocking the potential of deep counterfactual value networks. arXiv:2007.10442 [cs.AI] (20 July 2020).

[39] N. Brown, T. Sandholm, B. Amos, Combining deep reinforcement learning and search for imperfect-information games. *Proceedings of the Thirty-second Conference on Neural Information Processing Systems* (NeurIPS, 2018).

[40] P. I. Cowling, E. J. Powley, D. Whitehouse, Information set Monte Carlo tree search. IEEE Trans. Comput. Intell. AI Games 4, 120–143 (2012).

[41] J. Long, N. R. Sturtevant, M. Buro, T. Furtak, Understanding the success of perfect information Monte Carlo sampling in game tree search. *Proceedings of the Twenty-Fourth AAAI Conference on Artificial Intelligence, AAAI’10* (AAAI, 2010), p. 134–140.

[42] J. Nijssen, M. Winands, Monte Carlo tree search for the hide-and-seek game scotland yard. IEEE Trans. Comput. Intell. AI Games 4, 282–294 (2012).

[43] J. Nijssen, “Monte-Carlo tree search for multi-player games,” thesis, Maastricht University, Maastricht, The Netherlands (2012).

[44] J. Heinrich, D. Silver, Smooth UCT search in computer poker. *Proceedings of the 24th International Joint Conference on Artificial Intelligence* (IJCAI, 2015).

[45] V. Lisý, M. Lanctot, M. Bowling, Online Monte Carlo counterfactual regret minimizationfor search in imperfect information games. *Proceedings of the Fourteenth International Conference on Autonomous Agents and Multi-Agent Systems* (AAMAS, 2015), pp. 27–36.

[46] M. Lanctot, K. Waugh, M. Zinkevich, M. Bowling, Monte Carlo sampling for regret minimization in extensive games. *Advances in Neural Information Processing Systems 22* (NIPS, 2009), pp. 1078–1086.

[47] J. Heinrich, M. Lanctot, D. Silver, Fictitious self-play in extensive-form games. *Proceedings of the 32nd International Conference on Machine Learning* (ICML, 2015).

[48] M. Lanctot, V. Zambaldi, A. Gruslys, A. Lazaridou, K. Tuyls, J. Perolat, D. Silver, T. Graepel, A unified game-theoretic approach to multiagent reinforcement learning. *Advances in Neural Information Processing Systems* (NeurIPS, 2017).

[49] H. Li, K. Hu, S. Zhang, Y. Qi, L. Song, Double neural counterfactual regret minimization. *Proceedings of the Eighth International Conference on Learning Representations* (ICLR, 2019).

[50] N. Brown, A. Lerer, S. Gross, T. Sandholm, Deep counterfactual regret minimization. arXiv:1811.00164 [cs.AI] (1 November 2018).

[51] E. Steinberger, A. Lerer, N. Brown, DREAM: Deep regret minimization with advantage baselines and model-free learning. arXiv:2006.10410 [cs.LG] (18 June 2020).

[52] S. Srinivasan, M. Lanctot, V. Zambaldi, J. Pérolat, K. Tuyls, R. Munos, M. Bowling, Actor-critic policy optimization in partially observable multiagent environments. *Advances in Neural Information Processing Systems* (NeurIPS, 2018).

[53] E. Lockhart, M. Lanctot, J. Pérolat, J.-B. Lespiau, D. Morrill, F. Timbers, K. Tuyls, Computing approximate equilibria in sequential adversarial games by exploitability descent. *Proceedings of the 28th International Joint Conference on Artificial Intelligence* (IJCAI, 2019).

[54] D. Hennes, D. Morrill, S. Omidshafiei, R. Munos, J. Perolat, M. Lanctot, A. Gruslys, J.-B. Lespiau, P. Parmas, E. Duenez-Guzman, K. Tuyls, Neural replicator dynamics. *Proceedings of the International Conference on Autonomous Agents and Multiagent Systems* (AAMAS, 2020).

[55] A. Gruslys, M. Lanctot, R. Munos, F. Timbers, M. Schmid, J. Perolat, D. Morrill, V. Zambaldi, J.-B. Lespiau, J. Schultz, M. G. Azar, M. Bowling, K. Tuyls, The advantage regret-matching actor-critic. arXiv:2008.12234 [cs.AI] (27 August 2020).

[56] J. Perolat, R. Munos, J.-B. Lespiau, S. Omidshafiei, M. Rowland, P. Ortega, N. Burch, T. Anthony, D. Balduzzi, B. D. Vylder, G. Piliouras, M. Lanctot, K. Tuyls, From Poincaré recurrence to convergence in imperfect information games: Finding equilibrium via regularization. *Proceedings of the The Thirty-eighth International Conference on Machine Learning* (ICML, 2021).

[57] S. McAleer, J. Lanier, P. Baldi, R. Fox, XDO: A double oracle algorithm for extensive-form games. *Proceedings of the Thirty-fifth Conference on Neural Information Processing Systems* (NeurIPS, 2021).

[58] X. Feng, O. Slumbers, Z. Wan, B. Liu, S. M. McAleer, Y. Wen, J. Wang, Y. Yang, Neural auto-curricula in two-player zero-sum games. *Proceedings of the Thirty-fifth Conference on Neural Information Processing Systems* (NeurIPS, 2021).

[59] J. Perolat, B. D. Vylder, D. Hennes, E. Tarassov, F. Strub, V. de Boer, P. Muller, J. T. Connor, N. Burch, T. Anthony, S. McAleer, R. Elie, S. H. Cen, Z. Wang, A. Gruslys, A. Malysheva, M. Khan, S. Ozair, F. Timbers, T. Pohlen, T. Eccles, M. Rowland, M. Lanctot, J.-B. Lespiau, B. Piot, S. Omidshafiei, E. Lockhart, L. Sifre, N. Beauguerlange, R. Munos, D. Silver, S. Singh, D. Hassabis, K. Tuyls, Mastering the game of Stratego with model-free multiagent reinforcement learning. Science 378, 990–996 (2022). 36454847 10.1126/science.add4679

[60] T. Dash, S. N. Dambekodi, P. N. Reddy, A. Abraham, Adversarial neural networks for playing hide-and-search board game Scotland Yard. Neural Comput. Appl. 32, 3149–3164 (2020).

[61] J. N. Foerster, F. Song, E. Hughes, N. Burch, I. Dunning, S. Whiteson, M. Botvinick, M. Bowling, Bayesian action decoder for deep multi-agent reinforcement learning. arXiv:1811.01458 [cs.MA] (4 November 2019).

[62] S. Sokota, E. Lockhart, F. Timbers, E. Davoodi, R. D’Orazio, N. Burch, M. Schmid, M. Bowling, M. Lanctot, Solving common-payoff games with approximate policy iteration. *Proceedings of the Thirty-Fifth AAAI Conference on Artificial Intelligence* (AAAI, 2021).

[63] D. Strouse, K. R. McKee, M. Botvinick, E. Hughes, R. Everett, Collaborating with humans without human data. *Proceedings of the Thirty-fifth Conference on Neural Information Processing Systems* (NeurIPS, 2021).

[64] A. Bakhtin, N. Brown, E. Dinan, G. Farina, C. Flaherty, D. Fried, A. Goff, J. Gray, H. Hu, A. P. Jacob, M. Komeili, K. Konath, M. Kwon, A. Lerer, M. Lewis, A. H. Miller, S. Mitts, A. Renduchintala, S. Roller, D. Rowe, W. Shi, J. Spisak, A. Wei, D. Wu, H. Zhang, M. Zijlstra, Human-level play in the game of Diplomacy by combining language models with strategic reasoning. Science 378, 1067–1074 (2022). 36413172 10.1126/science.ade9097

[65] T. S. D. Team, Stockfish: Open source chess engine (2021); https://stockfishchess.org/.

[66] M. Johanson, Measuring the size of large no-limit poker games. arXiv:1302.7008 [cs.GT] (27 February 2013).

[67] Game of the year 1983: Scotland Yard; https://www.spiel-des-jahres.de/spiel-des-jahres-1983-scotland-yard/.

[68] F. Southey, M. Bowling, B. Larson, C. Piccione, N. Burch, D. Billings, C. Rayner, Bayes’ bluff: Opponent modelling in poker. *Proceedings of the Twenty-First Conference on Uncertainty in Artificial Intelligence* (UAI, 2005), pp. 550–558.

[69] A. Elo, The Rating of Chessplayers, Past and Present (Arco Pub., ed. 2, 1986).

[70] T. G. D. Team, Gnugo (2009); https://www.gnu.org/software/gnugo/.

[71] P. Baudis, J. loup Gailly, Lemonsqueeze, Pachi: Software for the board game of go / weiqi / baduk (2016); https://pachi.or.cz/.

[72] E. Jackson, Slumbot NL: Solving large games with counterfactual regret minimization using sampling and distributed processing. *Proceedings of the Computer Poker and Imperfect Information: Papers from the AAAI 2013 Workshop* (AAAI, 2013).

[73] E. Jackson, Slumbot github repository; https://github.com/ericgjackson/slumbot2017.

[74] N. Burch, M. Schmid, M. Moravčík,, M. Bowling, AIVAT: A new variance reduction technique for agent evaluation in imperfect information games. arXiv:1612.06915 [cs.AI] (20 December 2017).

[75] V. Lisý, M. Bowling, Equilibrium approximation quality of current no-limit poker bots. *Workshops at the Thirty-First AAAI Conference on Artificial Intelligence* (AAAI, 2017).

[76] R. B. Segal, On the scalability of parallel UCT. *CG’10: Proceedings of the 7th international Conference on Computers and Games* (Springer, 2010), pp. 36–47.

[77] P. J. Huber, Robust estimation of a location parameter. Ann. Math. Stat. 35, 73–101 (1964).

[78] N. Brown, T. Sandholm, Strategy-based warm starting for regret minimization in games. *Proceedings of the Thirtieth AAAI Conference on Artificial Intelligence* (AAAI, 2016), pp. 432–438.

[79] M. Lanctot, E. Lockhart, J.-B. Lespiau, V. Zambaldi, S. Upadhyay, J. Pérolat, S. Srinivasan, F. Timbers, K. Tuyls, S. Omidshafiei, D. Hennes, D. Morrill, P. Muller, T. Ewalds, R. Faulkner, J. Kramár, B. D. Vylder, B. Saeta, J. Bradbury, D. Ding, S. Borgeaud, M. Lai, J. Schrittwieser, T. Anthony, E. Hughes, I. Danihelka, J. Ryan-Davis, OpenSpiel: A framework for reinforcement learning in games. arXiv:1908.09453 [cs.LG] (26 August 2019).

[80] M. Šustr, V. Kovařík, V. Lisý, Monte Carlo continual resolving for online strategy computation in imperfect information games. *Proceedings of the 18th International Conference on Autonomous Agents and Multiagent Systems* (AAMAS, 2019), pp. 224–232.

