# Supplementary material for: Student of Games: A unified learning algorithm for both perfect and imperfect information games
Source: Sci Adv. 2023 Nov 15;9(46):eadg3256. doi: 10.1126/sciadv.adg3256 (PMC10651118; doi:10.1126/sciadv.adg3256)
Supplement: Supplementary file 1 — Supplementary Text Figs. S1 to S4 Tables S1 to S6 References [file sciadv.adg3256_sm.pdf]

Science Advances
AAAS

Supplementary Materials for

Student of Games: A unified learning algorithm for both perfect and imperfect information games

Martin Schmid *et al.*

Corresponding author: Michael Bowling, mbowling@ualberta.ca

Sci. Adv. **9**, eadg3256 (2023)
DOI: 10.1126/sciadv.adg3256

This PDF file includes:

Supplementary Text
Figs. S1 to S4
Tables S1 to S6
References

Supplementary Text

Student of Games Algorithm Details

Network Architecture and Optimization

Table S1 lists neural network architectures and input features used for each game. For chess and Go we use exactly the same architecture and inputs as used by AlphaZero (*6*). In poker and Scotland Yard we process concatenated belief and public state features by a MLP with ReLU activations.

The counterfactual value head is optimized by Huber loss (*77*), while policy for each information state i is optimized by KL divergence:

$$l(\mathbf{v}, \mathbf{p}, \mathbf{v}_{target}, \mathbf{p}_{target}) = w_v * l_{huber}(\mathbf{v}, \mathbf{v}_{target}) + w_p * \sum_i l_{KL}(\pi^i, \pi^i_{target})$$

where each head is weighted with the corresponding weight w_v and w_p. During training we smoothly decay the learning rate by a factor of d every T_{decay} steps. Formally learning rate α at training step t is defined as:

$$\alpha_t = \alpha_{init} * d^{t/T_{decay}}$$

When using the policy head's prediction as prior in PUCT formula the logits are processed with softmax with temperature T_{prior}. This can decrease weight of the prior in some games and encourage more exploration in the search phase.

Pseudocode

Here we provide pseudocode for the most important parts of the SoG algorithm. Algorithm 1 specifies GT-CFR, the core of SoG's sound game-theoretic search that scales to large perfect

information games introduced in Search via Growing-Tree CFR. Algorithm 2 presents how GT-CFR is used during self-play that generates training examples for the neural network, previously covered in Training Process. Hyperparameters used in self-play are specified in Table S2.

When SoG plays against an opponent, the search tree is rebuilt also for the opponent's actions (as discussed in Modified Continual Re-solving). This way, SoG reasons about the opponent's behavior since it directly influences the belief distribution for the current state β where SoG is to act.

Note that unlike AlphaZero, SoG currently starts its search procedure from scratch. That is, the previous computation only provides invariants for the next resolving step. AlphaZero rather warm-starts the MCTS process by initializing values and visit counts from the previous search. For SoG, this would also require warm-starting CFR. While possible (*78*), there is no warm-starting in the current implementation of SoG.

Implementation

SoG is implemented as a distributed system with decoupled actor and trainer jobs. Each actor runs several parallel games and the neural network evaluations are batched for better accelerator utilization. The networks were implemented using TensorFlow.

Poker Betting Abstraction

There are up to 20000 possible actions in no-limit Texas hold'em. To make the problem easier, AI agents are typically allowed to use only a small subset of these (*8, 37–39*). This process of selecting a set of allowed actions for a given poker state is called betting abstraction. Even using betting abstraction the players are able to maintain strong performance in the full game (*8, 37, 38*). Moreover, the local best response evaluation (*75*) suggests that there is not an easy exploit for such simplification as long as the agent is able to see full opponent actions (*8*).

Algorithm 1 Growing Tree CFR. Note that GT-CFR is logging all neural net queries it does since they might be used later in training.

procedure GT-CFR($\mathcal{L}^0, \beta, s, c$)

  ▷ $\mathcal{L}^0$ — a tree including β built as described in Modified Continual Re-solving.

  ▷ β — a public belief state under which the new nodes will be added.

  ▷ s, c — total number of expansion simulations and number of simulations per CFR update.

  for $i \in \{0, 1, \cdots, \frac{s}{\lceil c \rceil} - 1\}$ **do**

    CFR($\mathcal{L}^i, \lceil \frac{1}{c} \rceil$) ▷ Store average policy and counterfactual values in the tree.

    $\mathcal{L}^{i+1}$ GROW($\mathcal{L}^i$)

  end for

  ▷ Return counterfactual values and average policy from CFR and all NN calls.

  return $\mathbf{v}, \mathbf{p}, nn\_queries$

end procedure

procedure GROW($\mathcal{L}, \beta$)

  for $i \in \{0, 1, \cdots, \lceil c \rceil - 1\}$ **do**

    $path \leftarrow$ SAMPLEPATHDOWNTHETREE($\mathcal{L}, \beta$) ▷ The path starts at β.

    ADDTOPKCHILDREN($\mathcal{L}, path, k$)

    UPDATEVISITCOUNTSUP($\mathcal{L}, path$)

  end for

  return $\mathcal{L}$

end procedure

Algorithm 2 Sound Self-play

procedure SELFPLAY
    Get initial history state $w \leftarrow w^{INIT}$ and corresponding public state β
    ▷ Decide whether the game has to be played till the end.
    $do\_not\_resign \leftarrow$ coin flip with probability p_{no_resign}
    while w is not terminal AND played less than $moves_{max}$ **do**
        if chance acts in w **then**
            $a \leftarrow$ uniform random action.
        else
            ▷ SoG acts for all non-chance players.
            $v_w, \pi_w^{controller} \leftarrow$ SOGSELFPLAYCONTROLLER(w)
            if $v_w <$ $resign\_threshold$ AND $not(do\_not\_resign)$ **then**
                ▷ Don't waste compute on already decided game.
                return
            end if
            ▷ Mix controller's policy with uniform prior to encourage exploration.
            $\pi_w^{selfplay} \leftarrow (1-\epsilon)\cdot \pi_w^{controller} + \epsilon \cdot \pi^{uniform}$
            if moves played $< moves_{greedy\_after}$ **then**
                $a \leftarrow$ sample action from $\pi_w^{selfplay}$
            else
                $a \leftarrow \arg\max \pi_w^{selfplay}$
            end if
        end if
        $w \leftarrow$ apply action a on state w
    end while
    ▷ Sampling states with TD(1) targets.
    for each belief state $\beta \in$ played trajectory tr **do**
        if uniform random sample from unit interval $< p_{td1}$ **then**
            $\mathbf{v} \leftarrow$ outcome of tr assigned to information state visited in β
            $\mathbf{p} \leftarrow$ policy used in β
            replay_buffer.append($\langle \beta, (\mathbf{v}, \mathbf{p}) \rangle$)
        end if
    end for
end procedure

procedure SOGSELFPLAYCONTROLLER(w)

$\beta \leftarrow$ public state including w

$\mathcal{L} \leftarrow$ the tree including β built as described in Modified Continual Re-solving.

$\mathbf{v}, \mathbf{p} \leftarrow$ TRAINING-GT-CFR($\mathcal{L}$)

return $\mathbf{v}(w), \mathbf{p}(w)$

end procedure

procedure TRAINING-GT-CFR($\mathcal{L}$)

$\mathbf{v}, \mathbf{p}, nn\_queries \leftarrow$ GT-CFR($\mathcal{L}$)

queries $\leftarrow$ Pick on average q_{search} neural net queries β from $nn\_queries$.

queries_to_solve.extend(queries)

return $\mathbf{v}, \mathbf{p}$

end procedure

procedure QUERYSOLVER

for $\beta \leftarrow$ queries_to_solve.pop() **do**

$\mathbf{v}, \mathbf{p}, nn\_queries \leftarrow$ GT-CFR(β)

▷ Send the example to the trainer.

replay_buffer.append($\langle \beta, (\mathbf{v}, \mathbf{p}) \rangle$)

▷ Create recursive queries.

queries $\leftarrow$ Pick on average $q_{recursive}$ neural net queries β from $nn\_queries$.

queries_to_solve.extend(queries)

end for

end procedure

We use a betting abstraction in the Student of Games to speed up the training and simplify the learning task. Our agent's action set was limited to just 3 actions: fold (give up), check/call (match the current wager) and bet/raise (add chips to the pot). To improve generalization we used stochastic betting size similarly to ReBeL (*37*). The single allowed bet/raise size is randomly uniformly selected at the start of each poker hand from the interval $\langle 0.5, 1.0\rangle * pot\_size$. This amount is anecdotally similar to one used by human players and had good performance in our experiments. The same random selection was used in both training and evaluation.

As in (*37*), we have also randomly varied the stack size (number of chips available to the players) at the start of the each round during the training. This number stays fixed during evaluation.

Evaluation Details and Additional Experimental Results

Description of Leduc poker

Leduc is a simplified poker game with two rounds and a 6-card deck in two suits. Each player initially antes a single chip to play and obtains a single private card and there are three actions: fold, call and raise. There is a fixed bet amount of 2 chips in the first round and 4 chips in the second round, and a limit of two raises per round. After the first round, a single public card is revealed. A pair is the best hand, otherwise hands are ordered by their high card (suit is irrelevant). A player's reward is their gain or loss in chips after the game.

Reinforcement_Learning_and_Search_in_Imperfect_Information_Games

In this section, we provide some experimental results showing that common RL and widely-used search algorithms can produce highly exploitable strategies, even in small imperfect information games where exploitability is computable exactly. In particular, we show how exploitable Information Set Monte Carlo Tree Search is in Leduc poker, as well as three standard

RL algorithms (DQN, A2C and tabular Q-learning) in both Kuhn poker and Leduc poker using OpenSpiel (*79*). Results are presented in milli big blinds per hand (mbb/h), which corresponds to one thousandth of a chip for both games.

Information_Set_Monte_Carlo_Tree_Search

Information Set Monte Carlo Tree Search (IS-MCTS) is a search method that, at the start of each simulation, first samples a world state– consistent with the player's information state– and uses it for the simulation (*40*). Reward and visit count statistics are aggregated over information states so that players base their decisions only on their information states rather than on private information inaccessible to them.

Table S5 shows the exploitability of a policy obtained by running separate independent IS-MCTS searches from each information state in the game, over various parameter values. The lowest exploitability of IS-MCTS we found among this sweep was **465 mbb/h**.

Standard_RL_algorithms_in_Imperfect_Information_Games

As imperfect information games generally need stochastic policies to achieve an optimal strategy, one might wonder how exploitable standard RL algorithms are in this class of games. To test this, we trained three standard RL agents: DQN, policy gradient (A2C) and tabular Q-learning. We used MLP neural networks in DQN and A2C agents. Table S6 shows the hyper parameters we swept over to train these RL agents.

In Kuhn poker, the best performing A2C agent converges to exploitability of 52 mbb/h, and tabular Q-learning and DQN agents converge to around 250 mbb/h. Similarly, in Leduc poker, the best performing A2C agent converges to exploitability of 78 mbb/h, tabular Q-learning and DQN agents converge to about 1300 mbb/h and 900 mbb/h respectively. Fig. S4 shows the exploitability of RL agents in Kuhn poker and Leduc poker.

Proofs of Theorems

There are three substantive differences between the SoG algorithm and DeepStack. First, SoG uses a growing search tree, rather than using a fixed limited-lookahead tree. Second, the SoG search tree may depend on the observed chance events. Finally, SoG uses a continuous self-play training loop operating throughout the entire game, rather than the stratified bottom-up training process used by DeepStack. We address each of these differences below, in turn, after considering how to describe an approximate value function for search in imperfect information games.

Value Functions for Subgames

Like DeepStack, the SoG algorithm uses a value function, so the quality of its play depends on the quality of the value function. We will describe a value function in terms of its distance to a strategy with low regret. We start with some value and regret definitions that are better suited to subgames.

Consider some policy profile π which is a tuple containing a strategy for each player, public tree subgame S rooted at public state s_{pub} with player ranges $B_i[s_i \in \mathcal{S}_i(s_{\text{pub}})] := P_i(s_i|\pi)$. First, note that we can re-write counterfactual value v so that it depends only on B and π restricted to S, with no further dependence on π. Let s_i be a Player i information state in $\mathcal{S}_i(s_{\text{pub}})$, and q be the opponent of Player i, then:

$$\begin{aligned} v^{\mathbf{B},\pi^S}(s_i) &:= \sum_{h \in I(s_i)} \sum_{z \sqsupset h} B_q[s_q(h)] P_c(h) P(z|h,\pi^S) u_i(z) \\ &= \sum_{h \in I(s_i)} \sum_{z \sqsupset h} P_{-i}(h|\pi) P(z|h,\pi) u_i(z) = v^{\pi}(s_i) \end{aligned}$$

We can write several quantities in terms of the best-response value at information state s_i:

$$BV^{B,\pi^S}(s_i) := \max_{\pi_i^*} v^{B,\pi^S \leftarrow \pi_i^*}(s_i)$$

where $\pi \leftarrow \pi'$ is the policy profile constructed by replacing action probabilities in π with those in π'. The value function is a substitute for an entire subgame policy profile, so the regret we are interested in is player i's full counterfactual regret (*31*) at s_i, which considers all possible strategies within subgame S:

$$R^{\text{full}}_{s_i}(B, \pi^S) := BV^{B,\pi^S}(s_i) - v^{B,\pi^S}(s_i)$$

With these definitions in hand, we can now consider the quality of a value function f in terms of a regret bound ϵ and value error ξ. Recall that f maps ranges B and public state s_{pub} to approximate counterfactual values $\tilde{v}(s_i)$ for each player i.

First, we consider versions of the regret bound and value error which are parameterised by a strategy π. There is some associated bound $\epsilon(\pi)$ on the sum of regrets across all information states at any subgame, valid for both players.

$$\epsilon(\pi) := \max_B \max_{s_{\text{pub}}} \max_i \sum_{s_i \in \mathcal{S}_i(s_{\text{pub}})} R^{\text{full}}_{s_i}(B, \pi)$$

There is also some bound $\xi_f(\pi)$ on the distance between $f(s_{\text{pub}}, B)$ and the best-response values to π.

$$\xi_f(\pi) := \max_B \max_{s_{\text{pub}}} \max_i \sum_{s_i \in \mathcal{S}_i(s_{\text{pub}})} |f(s_{\text{pub}}, B)[s_i] - v^{B,\pi}(s_i)|$$

We then say that f has ϵ, ξ quality bounds if there exists some strategy π such that $\epsilon(\pi) \leq \epsilon$ and $\xi_f(\pi) \leq \xi$. As desired, if both ϵ and ξ are low then $f(s_{\text{pub}}, B)$ is a good approximation of the best-response values to a low-regret strategy, for a subgame rooted at s_{pub} with initial beliefs B.

The DeepStack algorithm (*8*) used a similar error metric for value functions, but only considered zero-regret strategies. We introduce a more complicated error measure because the space of values corresponding to low-regret strategies may be much larger than the space of

values corresponding to no-regret strategies. For example, consider the public subgame of a matching pennies game after the first player acts with the policy 0.501 heads, 0.499 tails. There are two first-player information states, from playing either heads or tails, with an empty first-player strategy as there are no further first player actions. Let us assume a value function f is returning the values $[0\ 0]$ for these two information states. How good is f, assuming we restrict our attention to this one subgame?

The unique zero-regret strategy for the second player is to play tails 100% of the time, resulting in first player counterfactual values of -1 for playing heads and 1 for playing tails. The error metric based on zero-regret strategies is therefore measuring $|f([0.501\ 0.499]) - [-1\ 1]|_1$, so that the DeepStack metric states that f has an error of 2. However, $[0\ 0]$ seems like a very reasonable choice: these are exactly the first player counterfactual values when the second player has a strategy of 0.5 heads, 0.5 tails, which has a regret of only 0.002 in this subgame. Rather than saying f is a poor quality value function with an error of 2 in a game with utilities in $[-1, 1]$, we can now say f is a great $0.002, 0$ value function which exactly describes a low-regret strategy.

The new quality metric also addresses an issue the old DeepStack metric had with discontinuities in the underlying 0-regret value functions. This means that the space of functions with a low DeepStack error metric may not be well suited for learning from data. Continuing with the previous example, if we shifted B slightly to be 0.499 heads and 0.501 tails for the first player, the unique 0-regret strategy in the subgame flips to playing tails 0% of the time, while the uniform random strategy is still a low-regret strategy for this subgame. In this example, a function can only have a low error with the DeepStack metric if it accurately predicts the values everywhere around the discontinuity at 0.5 heads and 0.5 tails, whereas the new metric can avoid this discontinuity by picking an $\epsilon > 0$. More generally, for any constant c, the objective $\epsilon + c\xi$ is a continuous function in B, making it a potentially more attractive learning target

than the discontinuous function defined by exact Nash equilibrium values, and which matches a learning procedure based on approximately solving example subgames.

Growing Trees

One major step in showing soundness of the SoG algorithm is demonstrating that Growing Tree CFR (GT-CFR) can approximately solve games. As a quick recap, GT-CFR is a variant of the CFR algorithm (*31*) that uses limited lookahead and a value function, storing values within a tree that grows over time, in a fashion similar to UCT (*27*). We use this algorithm as a component to solve the problems that the SoG algorithm sets up. At every non-terminal public leaf state s_{pub} of the lookahead tree, GT-CFR uses estimated counterfactual values $\tilde{v}$, generated from a value function $f(s_{\text{pub}}, B)$ with player ranges B induced by Bayes' rule at s_{pub} for the current policy profile π.

Like DeepStack, SoG has two steps which involve solving subgames of the original game. One of the steps is the re-solving step used to play through a game, where we solve a modified subgame based on constraints on opponent values and beliefs about our possible private information, in order to get our policy and new opponent values. The other step is only in the training loop, where we are solving a subgame with fixed beliefs for both players, in order to get values for both players. While the (sub)games for these two cases are slightly different, they are both well-formed games and we can find an approximate Nash equilibrium using GT-CFR.

When running GT-CFR, even though a policy is explicitly defined only at information states in the lookahead tree $\mathcal{L}$, at each iteration t there is implicitly some complete policy profile π^t. For any information state s in $\mathcal{L}$ which is not a leaf, $\pi^t(s)$ is explicitly defined by the regret-matching policy. For all other s – either a leaf of $\mathcal{L}$ or outside of the lookahead tree – $\pi^t(s)$ is defined by the ϵ-regret subgame policy profile $\pi^{*,S}$ associated with the value function's ϵ, ξ quality bounds. Note that this π^t only exists as a concept which is useful for theoretical

analysis: GT-CFR does not have access to the probabilities outside of its lookahead tree, only a noisy estimate of the associated counterfactual values provided by the value function.

Lemma 1. *Let* $\mathbf{p}$ *and* $\mathbf{q}$ *be vectors in* $[0,1]^n$*, and* $\mathbf{v}$ *and* $\mathbf{w}$ *be vectors in* $\mathbb{R}^n$ *such that* $\mathbf{v}[\mathrm{i}] > \mathbf{w}[\mathrm{i}]$ *for all* i*. Then* $\mathbf{p} \cdot \mathbf{v} - \mathbf{q} \cdot \mathbf{w} \leq \mathbf{1} \cdot (\mathbf{v} - \mathbf{w}) + \mathbf{p} \cdot \mathbf{w} - \mathbf{q} \cdot \mathbf{w}$

Proof.

$$\begin{aligned}
\mathbf{p} \cdot \mathbf{v} - \mathbf{q} \cdot \mathbf{w} &= \mathbf{p} \cdot \mathbf{v} - \mathbf{p} \cdot \mathbf{w} + \mathbf{p} \cdot \mathbf{w} - \mathbf{q} \cdot \mathbf{w} \\
&= \mathbf{p} \cdot (\mathbf{v} - \mathbf{w}) + \mathbf{p} \cdot \mathbf{w} - \mathbf{q} \cdot \mathbf{w} \\
&\leq \mathbf{1} \cdot (\mathbf{v} - \mathbf{w}) + \mathbf{p} \cdot \mathbf{w} - \mathbf{q} \cdot \mathbf{w}
\end{aligned}$$

□

Lemma 2. *Let* $\mathbf{p}$ *and* $\mathbf{q}$ *be vectors in* $[0,1]^n$*, and* $\mathbf{v}$ *and* $\mathbf{w}$ *be vectors in* $\mathbb{R}^n$ *such that* $\sum_{i=1}^{n} |\mathbf{v}[\mathrm{i}] - \mathbf{w}[\mathrm{i}]| \leq \xi$*. Then* $(\mathbf{p} - \mathbf{q}) \cdot \mathbf{v} \leq \xi + (\mathbf{p} - \mathbf{q}) \cdot \mathbf{w}$.

Proof.

$$\begin{aligned}
(\mathbf{p} - \mathbf{q}) \cdot \mathbf{v} &= (\mathbf{p} - \mathbf{q}) \cdot (\mathbf{v} - \mathbf{w}) + (\mathbf{p} - \mathbf{q}) \cdot \mathbf{w} \\
&\leq \sum_{i=1}^{n} |(\mathbf{p}[\mathrm{i}] - \mathbf{q}[\mathrm{i}])(\mathbf{v}[\mathrm{i}] - \mathbf{w}[\mathrm{i}])| + (\mathbf{p} - \mathbf{q}) \cdot \mathbf{w} \\
&\leq \sum_{i=1}^{n} |(\mathbf{v}[\mathrm{i}] - \mathbf{w}[\mathrm{i}])| + (\mathbf{p} - \mathbf{q}) \cdot \mathbf{w} \\
&\leq \xi + (\mathbf{p} - \mathbf{q}) \cdot \mathbf{w}
\end{aligned}$$

□

In GT-CFR, the depth-limited public tree used for search may change at each iteration. Let $\mathcal{L}^t$ be the public tree at time t. For any given tree $\mathcal{L}$, let $\mathcal{N}(\mathcal{L})$ be the interior of the

tree: all non-leaf, non-terminal public states. The interior of the tree is where regret matching is used to generate a policy, with regrets stored for all information states in interior public states. Let $\mathcal{F}(\mathcal{L})$ be the frontier of $\mathcal{L}$, containing non-terminal leaves, and $\mathcal{Z}(\mathcal{L})$ be the terminal public states. GT-CFR uses the value function at all public states in the frontier, receiving noisy estimates $\tilde{v}(s)$ of the true counterfactual values $v(s)$. We will distinguish between the true regrets R^T_s computed from the entire policy, and the regret $\tilde{R}^T_s$ computed using the estimated values $\tilde{v}(s)$. Given a sequence of trees across T iterations, let $\mathcal{T}_n(s_{\text{pub}})$ be the set of maximal length intervals $[a,b] \subseteq [1,T]$ where s_{pub} is in $\mathcal{N}(\mathcal{L}^t)$ for all $t \in [a,b]$. Let U be the maximum difference in counterfactual value between any two strategies, at any information state, and A be the maximum number of actions at any information state.

Lemma 3. *After running GT-CFR for T iterations starting at some initial public state s_0, using a value function with quality ϵ, ξ, regret for the strategies satisfies the bound*

$$\sum_{s_i \in \mathcal{S}_i(s_0)} R^T_{s_i} \leq \sum_{t=1}^{T} |\mathcal{F}(\mathcal{L}^t)|(\epsilon + \xi) + \sum_{s_{\text{pub}} \in \bigcup_{t=1}^{T} \mathcal{L}^t} |\mathcal{S}_i(s_{\text{pub}})| U \sqrt{A} \sum_{[a,b] \in \mathcal{T}_n(s_{\text{pub}})} \sqrt{|[a,b]|}$$

Proof. Starting with the definition of regret, and noting that regrets are independently maximised in a perfect recall game, we can rearrange terms to get

$$\begin{aligned}
\sum_{s_i \in \mathcal{S}_i(s_0)} R^T_{s_i} &= \sum_{s_i \in \mathcal{S}_i(s_0)} \max_{\pi_i^*} \sum_{t=1}^{T} v^{\pi^t \leftarrow \pi_i^*}(s_i) - \sum_{t=1}^{T} v^{\pi^t}(s_i)\Big) \\
&= \max_{\pi_i^*} \sum_{s_i \in \mathcal{S}_i(s_0)} \sum_{t=1}^{T} v^{\pi^t \leftarrow \pi_i^*}(s_i) - \sum_{t=1}^{T} v^{\pi^t}(s_i)\Big) \\
&= \max_{\pi_i^*} \sum_{t=1}^{T} \sum_{s_i \in \mathcal{S}_i(s_0)} \Big(v^{\pi^t \leftarrow \pi_i^*}(s_i) - v^{\pi^t}(s_i)\Big)
\end{aligned}$$

We can rewrite the counterfactual values of information state s_i in terms of the counterfactual value of leaves and terminals of the tree.

$$= \max_{\pi_i^*} \sum_{t=1}^{T} \left(\sum_{s_{\text{pub}} \in \mathcal{F}(\mathcal{L}^t)} \sum_{s_i \in \mathcal{S}_i(s_{\text{pub}})} \left(P_i(s_i|\pi_i^*) v^{\pi^t \leftarrow \pi_i^*}(s_i) - P_i(s_i|\pi^t) v^{\pi^t}(s_i) \right) \right.$$
$$\left. + \sum_{s_{\text{pub}} \in \mathcal{Z}(\mathcal{L}^t)} \sum_{z \in I(s_{\text{pub}})} \left(P_i(z|\pi^t \leftarrow \pi_i^*) v^{\pi^t}(z) - P_i(z|\pi^t) v^{\pi^t}(z) \right) \right) \quad (1)$$

Examining part of the first term inside the sum, we can independently maximise the counterfactual values at each information state s_i. As above, this is equivalent to maximising at public state s_{pub}.

$$\sum_{s_i \in \mathcal{S}_i(s_{\text{pub}})} \left(P_i(s_i|\pi_i^*) v^{\pi^t \leftarrow \pi_i^*}(s_i) - P_i(s_i|\pi^t) v^{\pi^t}(s_i) \right)$$
$$\leq \sum_{s_i \in \mathcal{S}_i(s_{\text{pub}})} \max_{\pi^{**}} \left(P_i(s_i|\pi_i^*) v^{\pi^t \quad \pi_i^{**}}(s_i) - P_i(s_i|\pi^t) v^{\pi^t}(s_i) \right)$$
$$= \max_{\pi^{**}} \sum_{s_i \in \mathcal{S}_i(s_{\text{pub}})} \left(P_i(s_i|\pi_i^*) v^{\pi^t \quad \pi_i^{**}}(s_i) - P_i(s_i|\pi^t) v^{\pi^t}(s_i) \right)$$

Given that we individually maximised over each minuend, we satisfy the requirements of Lemma 1. We can then use the value function quality bounds.

$$\leq \max_{\pi^{**}} \sum_{s_i \in \mathcal{S}_i(s_{\text{pub}})} \left(v^{\pi^t \leftarrow \pi_i^{**}}(s_i) - v^{\pi^t}(s_i) \right)$$
$$+ \sum_{s_i \in \mathcal{S}_i(s_{\text{pub}})} \left(P_i(s_i|\pi_i^*) v^{\pi^t}(s_i) - P_i(s_i|\pi^t) v^{\pi^t}(s_i) \right)$$
$$\leq \epsilon + \sum_{s_i \in \mathcal{S}_i(s_{\text{pub}})} \left(P_i(s_i|\pi_i^*) v^{\pi^t}(s_i) - P_i(s_i|\pi^t) v^{\pi^t}(s_i) \right)$$

Up to this point, we have used the true counterfactual values for the current policy profile. At leaves, however, GT-CFR only has access to the value function's noisy estimates of the true values. Applying Lemma 2, we get

$$\leq \epsilon + \xi + \sum_{s_i \in \mathcal{S}_i(s_{\text{pub}})} \left(P_i(s_i|\pi_i^*) \tilde{v}^{\pi^t}(s_i) - P_i(s_i|\pi^t) \tilde{v}^{\pi^t}(s_i) \right)$$

Placing this back into Equation 1 and collecting ϵ and ξ terms, we have

$$\sum_{s_i \in \mathcal{S}_i(s_0)} R^T_{s_i} \leq \sum_{t=1}^{T} |\mathcal{F}(\mathcal{L}^t)|(\epsilon + \xi) + \max_{\pi_i^*} \sum_{t=1}^{T} \left(\sum_{s_{\text{pub}} \in \mathcal{F}(\mathcal{L}^t)} \sum_{s_i \in \mathcal{S}_i(s_{\text{pub}})} \left(P_i(s_i|\pi_i^*)\tilde{v}^{\pi^t}(s_i) - P_i(s_i|\pi^t)\tilde{v}^{\pi^t}(s_i) \right) + \sum_{s_{\text{pub}} \in \mathcal{Z}(\mathcal{L}^t)} \sum_{z \in I(s_{\text{pub}})} \left(P_i(z|\pi^t \leftarrow \pi_i^*)v^{\pi^t}(z) - P_i(z|\pi^t)v^{\pi^t}(z) \right) \right)$$

We can rearrange the sums to consider the regret contribution for each public state

$$= \sum_{t=1}^{T} |\mathcal{F}(\mathcal{L}^t)|(\epsilon + \xi) + \max_{\pi_i^*} \sum_{s_{\text{pub}} \in \bigcup_{t=1}^{T} \mathcal{L}^t} \left(\sum_{t \text{ s.t. } s_{\text{pub}} \in \mathcal{F}(\mathcal{L}^t)} \sum_{s_i \in \mathcal{S}_i(s_{\text{pub}})} \left(P_i(s_i|\pi_i^*)\tilde{v}^{\pi^t}(s_i) - P_i(s_i|\pi^t)\tilde{v}^{\pi^t}(s_i) \right) + \sum_{t \text{ s.t. } s_{\text{pub}} \in \mathcal{Z}(\mathcal{L}^t)} \sum_{z \in I(s_{\text{pub}})} \left(P_i(z|\pi^t \leftarrow \pi_i^*)v^{\pi^t}(z) - P_i(z|\pi^t)v^{\pi^t}(z) \right) \right)$$

As before we can use Lemma 1 to separate out regrets at the interior states in $\mathcal{N} := \mathcal{F}(\mathcal{N}(\bigcup_{t=1}^{T} \mathcal{L}^t))$, which always depend only on leaves and terminals. Let $\mathcal{L}'^{,t}$ be $\mathcal{L}^t$ minus all public states in $\mathcal{N}$ and any successor states.

$$\leq \sum_{t=1}^{T} |\mathcal{F}(\mathcal{L}^t)|(\epsilon + \xi) + \sum_{s_{\text{pub}} \in \mathcal{N}} \sum_{[a,b] \in \mathcal{T}_n(s_{\text{pub}})} \sum_{s_i \in s_{\text{pub}}} \tilde{R}^{a,b}_{s_i} + \max_{\pi_i^*} \sum_{s_{\text{pub}} \in \bigcup_{t=1}^{T} \mathcal{L}'^{,t}} \left(\sum_{t \text{ s.t. } s_{\text{pub}} \in \mathcal{F}(\mathcal{L}'^{,t})} \sum_{s_i \in \mathcal{S}_i(s_{\text{pub}})} \left(P_i(s_i|\pi_i^*)\tilde{v}^{\pi^t}(s_i) - P_i(s_i|\pi^t)\tilde{v}^{\pi^t}(s_i) \right) + \sum_{t \text{ s.t. } s_{\text{pub}} \in \mathcal{Z}(\mathcal{L}'^{,t})} \sum_{z \in I(s_{\text{pub}})} \left(P_i(z|\pi^t \leftarrow \pi_i^*)v^{\pi^t}(z) - P_i(z|\pi^t)v^{\pi^t}(z) \right) \right)$$

Note that the states which were separated out are now effectively terminals in smaller trees.

We

can repeat this process until regrets for all public states have been separated out.

$$\leq \sum_{t=1}^{T} |\mathcal{F}(\mathcal{L}^t)|(\epsilon + \xi) + \sum_{s_{\text{pub}} \in \bigcup_{t=1}^{T} \mathcal{L}^t} \sum_{[a,b] \in \mathcal{T}_n(s_{\text{pub}})} \sum_{s_i \in s_{\text{pub}}} \tilde{R}_{s_i}^{a,b}$$

Finally, from bounds on regret-matching (*32*),

$$\leq \sum_{t=1}^{T} |\mathcal{F}(\mathcal{L}^t)|(\epsilon + \xi) + \sum_{s_{\text{pub}} \in \bigcup_{t=1}^{T} \mathcal{L}^t} |\mathcal{S}_i(s_{\text{pub}})| U \sqrt{A} \sum_{[a,b] \in \mathcal{T}_n(s_{\text{pub}})} \sqrt{|[a,b]|}$$

□

Note that the form of Lemma 3 implies that regret might not be sub-linear if public states are repeatedly added and removed from the lookahead tree. If we only add states and never remove them, however, we get a standard CFR regret bound plus error terms for the value function.

Theorem 3. *Assume the conditions of Lemma 3 hold, and public states are never removed from the lookahead tree. Then*

$$R_i^{T,\mathit{full}} \leq \sum_{t=1}^{T} |\mathcal{F}(\mathcal{L}^t)|(\epsilon + \xi) + \sum_{s_{\text{pub}} \in \mathcal{N}(\mathcal{L}^T)} |\mathcal{S}_i(s_{\text{pub}})| U \sqrt{AT}$$

Proof. This follows from Lemma 3, noting that the interior of $\mathcal{L}^t$ monotonically grows over time. □

Self-play Values as Re-solving Constraints

By using a value network in solving, we lose the ability to compute our opponent's counterfactual best response values to our average strategy (*80*). It is easy to track the opponent's average self-play value across iterations of a CFR variant, but using these values as re-solving constraints does not trivially lead to a bound on exploitability for the re-solved strategy. We show here that average CFR self-play values lead to reasonable, controllable error bounds in the context of continual re-solving. We will use $(x)^+$ to mean $\max\{x, 0\}$. For simplicity, we

will also assume that the subgame that is being re-solved is in the GT-CFR lookahead tree for all iterations.

Theorem 4. *Assume we have some average strategy $\bar{\pi}$ generated by T iterations of GT-CFR solver using a value function with quality ϵ, ξ, with final lookahead tree $\mathcal{L}^T$ where public states were never removed from the lookahead tree, and a final average regret R_i^T for the player of interest. Further assume that we have re-solved some public subgame S rooted public state s_{pub}, using the average counterfactual values $\bar{v}(s_o) := \frac{1}{T}\sum_{t=1}^{T} v^{\pi^t}(s_o)$ as the opt-out values in the re-solving gadget. Let π^S be the strategy generated from the re-solving game, with some player and opponent average regrets $\bar{R}_i^S$ and $\bar{R}_o^S$, respectively. Then*

$$\begin{aligned} BV_o^{\bar{\pi}\leftarrow\pi^S} - BV_o^{\bar{\pi}} \leq & (\bar{R}_o^S)^+ + (\bar{R}_i^S)^+ + \bar{R}_i \\ & + 2(\max_t |\mathcal{F}(\mathcal{L}^t_{s_{\text{pub}}})|(\epsilon + \xi) + \sum_{s_{\text{pub}} \in \mathcal{N}(\mathcal{L}^T_{s_{\text{pub}}})} |\mathcal{S}_i(s_{\text{pub}})| U \sqrt{\frac{A}{T}}) \end{aligned}$$

Proof. The general outline of the proof has two parts, both asking the question "how much can the opponent best response value increase?" As in Lemma 4 of (*8*), we can consider breaking the error in re-solving opt-out values into separate underestimation and overestimation terms. The first part of this proof is a bound that takes into account the re-solving solution quality, and how much the average values underestimate the best response to the average. This underestimation is bounded by the opponent regret at a subgame, which requires the solving algorithm to have low regret everywhere in the game: low regret for the opponent does not directly imply that the opponent has low regret in portions of the game that they do not play. The second part of the proof is placing a bound on the overestimation, using the player's regret rather than the opponent's regret.

We start by noting that from the opponent player o's point of view, we can replace an information set s_o with a terminal that has utility $BV^{\bar{\pi}}(s_o)$, and the best response utility $\text{BV}_o^{\bar{\pi}, s_o \leftarrow BV^{\bar{\pi}}(s_o)}$

in this modified game will be equal to $\text{BV}_o^{\bar{\pi}}$. We can extend this to the entire subgame S, replacing each s_o with a terminal giving the opponent the best response value: $\text{BV}_o^{\bar{\pi},S\leftarrow BV^{\bar{\pi}}(S)} = \text{BV}_o^{\bar{\pi}}$. Using this notation, we can rewrite $\text{BV}_o^{\bar{\pi}\leftarrow\pi^S}$:

$$\begin{aligned} &\text{BV}_o^{\bar{\pi}\leftarrow\pi^S} - \text{BV}_o^{\bar{\pi}} \\ =&\text{BV}_o^{\bar{\pi},S\leftarrow BV^{\pi^S}(S)} - \text{BV}_o^{\bar{\pi}} \end{aligned}$$

Next, note that $BV^{\pi^S}(s_o)$, the opponent's counterfactual best response to the re-solved subgame strategy π^S at any s_o at the root of S, is no greater than the value of $\max\{BV^{\pi^S}(s_o), \bar{v}(s_o)\}$, the value of s_o within the re-solving game before the gadget where the opponent has decision to opt-out for a fixed value $\bar{v}(s_o)$. That is, adding an extra opponent action which terminates the game never decreases the opponent's best response utility. Extending this to the entire subgame S again, we get

$$\begin{aligned} &\text{BV}_o^{\bar{\pi},S\leftarrow BV^{\pi^S}(S)} - \text{BV}_o^{\bar{\pi}} \\ \leq&\text{BV}_o^{\bar{\pi},S\leftarrow \max\{BV^{\pi^S}(S),\bar{v}\}} - \text{BV}_o^{\bar{\pi}} \end{aligned} \tag{2}$$

From Lemma 1 of (*8*), the game value of a re-solving game with opt-out values $\bar{v}(s_o)$ is $U_{\bar{v},\bar{\pi}}^S + \sum_{s_o\in\mathcal{S}_o(s_{\text{pub}})} \bar{v}(s_o)$, for some underestimation error on the opt-out values that is given by

$$U_{\bar{v},\bar{\pi}}^S := \min_{\pi^{*S}} \sum_{s_o\in\mathcal{S}_o(s_{\text{pub}})} (BV^{\bar{\pi}\leftarrow\pi^{*S}}(s_o) - \bar{v}(s_o))^+$$

Given the re-solving regrets, we have $\text{BV}_o^{\pi^S} \leq (\bar{R}_o^S)^+ + (\bar{R}_i^S)^+ + U_{\bar{v},\bar{\pi}}^S + \sum_{s_o\in\mathcal{S}_o(s_{\text{pub}})} \bar{v}(s_o)$. Because $\text{BV}_o^{\bar{\pi},s_o\leftarrow w+\epsilon} \leq \text{BV}_o^{\bar{\pi},s_o\leftarrow w} + \epsilon$ for $\epsilon \geq 0$, we can use this inequality to update Equation 2. That is, there is some per-information-set values ϵ such that $BV^{\pi^S}(\tilde{S}) = \bar{v}(\cdot) + \epsilon$ and $\epsilon \cdot \mathbf{1} \leq (\bar{R}_o^S)^+ + (\bar{R}_i^S)^+ + U_{\bar{v},\bar{\pi}}^S$, so that

$$
\begin{aligned}
&\mathrm{BV}_o^{\bar{\pi},S\leftarrow\max\{BV^{\pi^S}(S),\bar{v}\}} - \mathrm{BV}_o^{\bar{\pi}} \\
=&\mathrm{BV}_o^{\bar{\pi},S\leftarrow\bar{v}+\epsilon} - \mathrm{BV}_o^{\bar{\pi}} \\
\leq&\mathrm{BV}_o^{\bar{\pi},S\leftarrow\bar{v}} + \epsilon\cdot\mathbf{1} - \mathrm{BV}_o^{\bar{\pi}} \\
\leq&\mathrm{BV}_o^{\bar{\pi},S\leftarrow\bar{v}} + (\bar{R}_o^S)^+ + (\bar{R}_i^S)^+ + U_{\bar{v},\bar{\pi}}^S - \mathrm{BV}_o^{\bar{\pi}}
\end{aligned}
\tag{3}
$$

Looking at $U_{\bar{v},\bar{\pi}}^S$, we note that this minimum is no greater than the case when $\pi^* = \bar{\pi}$. The difference $BV^{\bar{\pi}\leftarrow\pi^{*S}}(s_o) - \bar{v}(s_o)$ is the average full counterfactual regret R_{s_o} of strategy $\bar{\pi}$ at s_o. Restricting our attention to $\mathcal{L}_{s_{\text{pub}}}^t$, the portion of the lookahead tree restricted to s_{pub} and its descendants, Theorem 3 gives us a bound on $U_{\bar{v},\bar{\pi}}^S$ and we can update Equation 3

$$
\begin{aligned}
&\mathrm{BV}_o^{\bar{\pi},S\leftarrow\bar{v}} + (\bar{R}_o^S)^+ + (\bar{R}_i^S)^+ + U_{\bar{v},\bar{\pi}}^S - \mathrm{BV}_o^{\bar{\pi}} \\
\leq&\mathrm{BV}_o^{\bar{\pi},S\leftarrow\bar{v}} - \mathrm{BV}_o^{\bar{\pi}} \\
&+ (\bar{R}_o^S)^+ + (\bar{R}_i^S)^+ \max_t |\mathcal{F}(\mathcal{L}_{s_{\text{pub}}}^t)|(\epsilon+\xi) + \sum_{s_{\text{pub}}\in\mathcal{N}(\mathcal{L}_{s_{\text{pub}}}^T)} |\mathcal{S}_i(s_{\text{pub}})| U\sqrt{\frac{A}{T}}
\end{aligned}
\tag{4}
$$

Looking at just the difference in opponent counterfactual best response values, we can again get an upper bound by giving the opponent the choice at all information sets at the root of subgame S of playing a best response against the unmodified strategy $\bar{\pi}$ to get value $BV^{\bar{\pi}}(S)$, or opting out to get value $\bar{v}$.

$$
\begin{aligned}
&\text{BV}_o^{\bar{\pi},S\leftarrow\bar{v}} - \text{BV}_o^{\bar{\pi}} \\
\leq&\text{BV}_o^{\bar{\pi},S\leftarrow\max\{BV^{\bar{\pi}}(S),\bar{v}\}} - \text{BV}_o^{\bar{\pi}} \\
=&(\text{BV}_o^{\bar{\pi},S\leftarrow\max\{BV^{\bar{\pi}}(S),\bar{v}\}} - \bar{v}_o) - (\text{BV}_o^{\bar{\pi}} - \bar{v}_o) \\
\leq&(\text{BV}_o^{\bar{\pi},S\leftarrow\max\{BV^{\bar{\pi}}(S),\bar{v}\}} - \bar{v}_o) - (\text{BV}_o^{\pi^*} - \bar{v}_o) \\
=&(\text{BV}_o^{\bar{\pi},S\leftarrow\max\{BV^{\bar{\pi}}(S),\bar{v}\}} - \bar{v}_o) - (v_o^{\pi^*} - \bar{v}_o) \\
=&(\text{BV}_o^{\bar{\pi},S\leftarrow\max\{BV^{\bar{\pi}}(S),\bar{v}\}} - \bar{v}_o) + (v_i^{\pi^*} - \bar{v}_i) \\
\leq&(\text{BV}_o^{\bar{\pi},S\leftarrow\max\{BV^{\bar{\pi}}(S),\bar{v}\}} - \bar{v}_o) + (\text{BV}_i^{\bar{\pi}} - \bar{v}_i) \\
=&(\text{BV}_o^{\bar{\pi},S\leftarrow\max\{BV^{\bar{\pi}}(S),\bar{v}\}} - \bar{v}_o) + \bar{R}_i
\end{aligned} \tag{5}
$$

The difference of the first two terms is the regret in the opt-out game game described above, where we have lifted each iteration strategy π^t into this game by never selecting the opt-out choice. Consider the immediate counterfactual regret $\tilde{R}^T(s_o)$ in this situation for any information state s_o in this augmented game. Writing this in terms of the original immediate counterfactual regret $R^T(s_o)$ and the opt-out value, we get

$$
\begin{aligned}
\tilde{R}^T(s_o) &= \max\{T(\bar{v}[s_o] - \bar{v}[s_o]), R^T(s_o)\} \\
&= (R^T(s_o))^+
\end{aligned}
$$

Because the positive immediate regret in the opt-out game is the same as the positive regret in the original game, we can use the Theorem 3 bound, which is composed from immediate regrets. Putting this together with Equation 4 and Equation 5, we get

$$
\begin{aligned}
&\text{BV}_o^{\bar{\pi}\leftarrow\pi^S} - \text{BV}_o^{\bar{\pi}} \\
\leq&(\bar{R}_o^S)^+ + (\bar{R}_i^S)^+ + \bar{R}_i \\
&+ 2(\max_t |\mathcal{F}(\mathcal{L}^t_{s_{\text{pub}}})|(\epsilon + \xi) + \sum_{s_{\text{pub}} \in \mathcal{N}(\mathcal{L}^T_{s_{\text{pub}}})} |\mathcal{S}_i(s_{\text{pub}})| U \sqrt{\frac{A}{T}})
\end{aligned}
$$

□

Continual Re-solving

Continual re-solving puts GT-CFR together with re-solving the previously solved subgame. A bound on final solution quality follows directly from applications of Theorem 3 and Theorem 4.

Theorem 5. *Assume we have played a game using continual re-solving, with one initial solve and D re-solving steps. Each solving or re-solving step finds an approximate Nash equilibrium through T iterations of GT-CFR using a value function with quality ϵ, ξ, public states are never removed from the lookahead tree, the maximum interior size $\sum_{s_{\text{pub}} \in \mathcal{N}(\mathcal{L}^T)} |\mathcal{S}_i(s_{\text{pub}})|$ of all lookahead trees is bounded by N, the sum of frontier sizes across all lookahead trees is bounded by F, the maximum number of actions at any information sets is A, and the maximum difference in values between any two strategies is U. The exploitability of the final strategy is then bounded by $(5D+2)\left(F(\epsilon+\xi) + NU\sqrt{\frac{A}{T}}\right)$.*

Proof. The exploitability EXP_0 of the player's initial strategy from the original solve is bounded by the sum of the regrets for both players. Theorem 3 provides regret bounds for GT-CFR, so

$$\text{EXP}_0 \leq 2 \quad F(\epsilon+\xi) + NU\sqrt{\frac{A}{T}}\Bigg)$$

Each subsequent re-solve is operating on the strategy of the previous step, using the average values for the opt-out values. That is, the first re-solve will be updating the strategy from the initial solve, the second re-solve will be updating the subgame strategy from the first re-solve, and so on. Theorem 4 provides a bound on how much the exploitability increases after each re-solving step, with Theorem 3 providing the necessary regret bounds

$$\begin{aligned} \text{EXP}_d \leq & \text{EXP}_{d-1} + (\bar{R}^S_o)^+ + (\bar{R}^S_i)^+ + \bar{R}_i + 2 \quad F(\epsilon+\xi) + NU\sqrt{\frac{A}{T}}\Bigg) \\ \leq & \text{EXP}_{d-1} + 5 \quad F(\epsilon+\xi) + NU\sqrt{\frac{A}{T}}\Bigg) \end{aligned}$$

Unrolling for D re-solving steps leads to the final bound. □

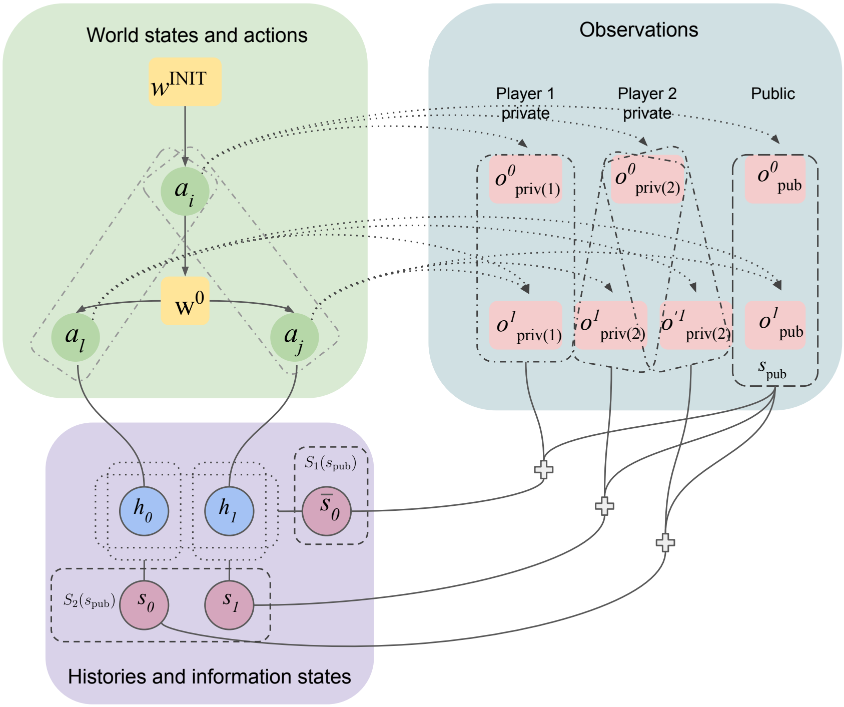

Figure S1: An example of a Factored-Observation Stochastic Game (FOSG). This figure presents the visual view of notation from Background and Terminology. In this example the game starts in w^{init} which is the complete state of the environment containing private information for both players. After playing action a_i the state moves to w^0 where there are two possible actions. Each action emits private and public observations. In this example, actions a_j and a_l emit the same private observation $o^1_{\text{priv}(1)}$ for player 1, therefore they cannot distinguish which action happened. On the other hand, player 2 has different observations $o^1_{\text{priv}(2)}$ and $o'^1_{\text{priv}(2)}$ for each of the actions, therefore they have more information about the state of the environment than player 1. The sequence of public observations shared by both players information is denoted as s_{pub}. Both sequences of actions and factored observations meet in the final 'Histories and information states' view. The two possible action sequences are represented by histories h_0 and h_1, where $h_0 = (a_i, a_l)$, $h_1 = (a_i, a_j)$. Since both actions a_l and a_j result in the same observation for player 1, they cannot tell which one of the histories happened and his information state $\bar{s}_0$ contains them both. This is not the case for player 2, who can separate the histories, and each of his information states s_0 and s_1 contains just one history.

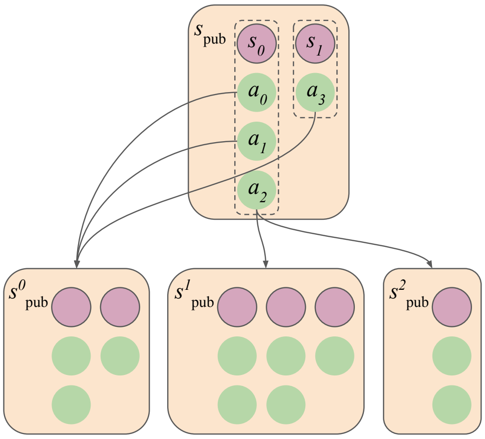

Figure S2: An example of a public tree. The public tree provides different view of the FOSG. In this example actions a_0 and a_1 emit the same public observation and therefore they lead to the same public tree node s^0_{pub}. On the other hand, action a_2 can lead to multiple possible states: for instance when a detective in Scotland Yard moves to a location the game can either 1) end because Mr. X was there and he was caught or 2) it continues because he was in a different station.

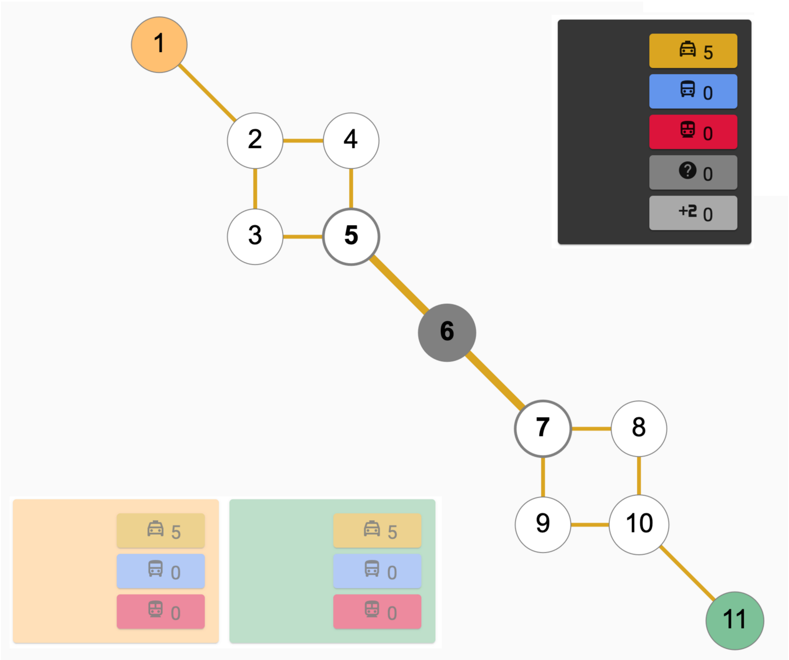

Figure S3: Initial situation on the glasses map for Scotland Yard. Mr. X starts at station 6 while the two detectives start at stations 1 and 11. All of them have 5 taxi cards (all edges in this map are of the same type) and the game is played for 5 rounds.

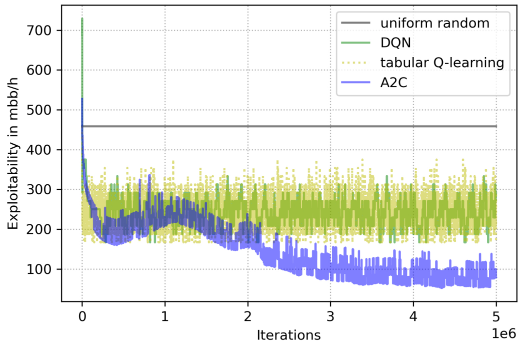

(**A**) Exploitability in 2-player Kuhn poker

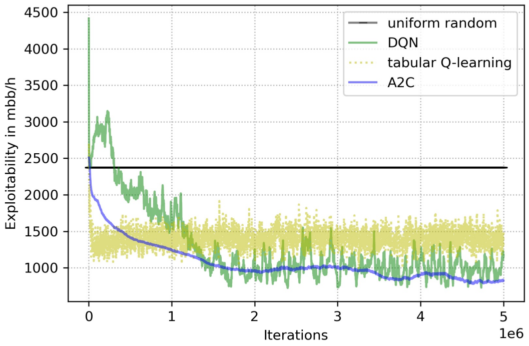

(**B**) Exploitability in 2-player Leduc poker

Figure S4: Comparing performance of DQN, A2C, tabular Q-learning and uniform random policy in (A) Kuhn poker and (B) Leduc poker.

Game	Architecture	Belief features	Public state features
Chess	ResNet	Redundant — there is no uncertainty over players state.	One 8x8 plane for each piece type (6) of each player (2) and repetitions planes (2) for last eight moves + scalar planes (7), 119 8x8 planes in total.
Go	ResNet	Redundant	One 19x19 plane for stones of each player (2) for last eight moves plus a single plane encoding player to act, 17 19x19 planes in total.
Poker	MLP 6 x 2048	1326 (possible private card combinations) * 2 (num of players).	N hot encoding of board cards (52) + commitment of each player normalized by his stack (2) + 1 hot encoding of who acts next, including chance player (3).
Scotland Yard	MLP 6 x 512	1 (detectives' position is always certain) + 199 (possible Mr X's position).	1 hot encoding of position of each detective (5*199) + cards of each detective (5*3) + cards of Mr X (5) + who is playing next (6) + was double move just used (1) + how many rounds were played (1).

Table S1: A neural network architecture and features used for each game.

Hyperparam	Symbol	Chess	Go	Scot. Yard	HUNL
Batch size		2048	2048	1024	1024
Optimizer		sgd	sgd	sgd	adam
Initial learning rate (LR)	α_{init}	0.1	0.02	0.1	0.0001
LR decay steps	T_{decay}	40k	200k	2M	2M
LR decay rate	d	0.8	0.1	0.5	0.5
Policy head weight	w_p	1	1	0.05	0.01
Value head weight	w_v	0.25	0.5	1	1
Replay buffer size		50M	50M	1M	1M
Max grad updates per example		1	0.2	5	10
TD(1) target sample probability	p_{td1}	0	0.2	0	0
Queries per search	q_{search}	1	0	0.3	0.9
Recursive queries per search	$q_{recursive}$	0.2	0	0.1	0.1
Self-play uniform policy mix	ϵ	0	0	0	0.1
Resign enabled		True	True	False	False
Resign threshold	$resign\_threshold$	-0.9	-0.9	-	-
Min ratio of games without resign	p_{no_resign}	0.2	0.2	-	-
Greedy play after move	$moves_{greedy\_after}$	30	30	never	never
Max moves in one episode	$moves_{max}$	512	722	unlim.	unlim.
Prior softmax temperature	T_{prior}	1.5	1.5	1	1

Table S2: Hyperparameters for each game.

Agent	Rel. Elo
AlphaZero(s=16k, t=800k)	+3139
AlphaZero(s=16k, t=400k)	+3021
AlphaZero(s=8k, t=800k)	+2875
AlphaZero(s=8k, t=400k)	+2801
AlphaZero(s=4k, t=800k)	+2643
AlphaZero(s=16k, t=200k)	+2610
AlphaZero(s=4k, t=400k)	+2584
AlphaZero(s=2k, t=800k)	+2451
AlphaZero(s=8k, t=200k)	+2428
AlphaZero(s=2k, t=400k)	+2353
AlphaZero(s=4k, t=200k)	+2234
AlphaZero(s=800, t=800k)	+2099
AlphaZero(s=16k, t=100k)	+2088
AlphaZero(s=2k, t=200k)	+2063
AlphaZero(s=800, t=400k)	+2036
**SoG(s=16k, c=10)**	**+1970**
AlphaZero(s=8k, t=100k)	+1940
**SoG(s=8k, c=10)**	**+1902**
AlphaZero(s=800, t=200k)	+1812
**SoG(s=4k, c=10)**	**+1796**
AlphaZero(s=4k, t=100k)	+1783
**SoG(s=2k, c=10)**	**+1672**
AlphaZero(s=2k, t=100k)	+1618
**SoG(s=800, c=1)**	**+1426**
AlphaZero(s=800, t=100k)	+1360
Pachi(s=100k)	+869
Pachi(s=10k)	+231
GnuGo(l=10)	+0

Table S3: Full Go results (Non Recursive Queries). Elo of GnuGo with a single thread and 100ms thinking time was set to be 0. AlphaZero(s=16k, t=800k) refers to 16000 search simulations after 800000 training steps.

Agent	Rel. Elo
AlphaZero(s=16k, t=800k)	+3431
AlphaZero(s=16k, t=400k)	+3319
AlphaZero(s=8k, t=800k)	+3169
AlphaZero(s=8k, t=400k)	+3093
AlphaZero(s=4k, t=800k)	+2933
AlphaZero(s=16k, t=200k)	+2899
AlphaZero(s=4k, t=400k)	+2880
AlphaZero(s=2k, t=800k)	+2745
AlphaZero(s=8k, t=200k)	+2712
AlphaZero(s=2k, t=400k)	+2643
AlphaZero(s=4k, t=200k)	+2509
AlphaZero(s=800, t=800k)	+2394
AlphaZero(s=16k, t=100k)	+2391
AlphaZero(s=2k, t=200k)	+2348
AlphaZero(s=800, t=400k)	+2315
AlphaZero(s=8k, t=100k)	+2240
AlphaZero(s=800, t=200k)	+2105
AlphaZero(s=4k, t=100k)	+2078
**SoG(s=16k, c=10)**	**+2025**
**SoG(s=8k, c=10)**	**+1937**
AlphaZero(s=2k, t=100k)	+1928
**SoG(s=4k, c=10)**	**+1838**
**SoG(s=2k, c=10)**	**+1766**
AlphaZero(s=800, t=100k)	+1644
**SoG(s=800, c=1)**	**+1579**
Pachi(s=100k)	+958
Pachi(s=10k)	+227
GnuGo(l=10)	+0

Table S4: Full Go results (Recursive Queries). Elo of GnuGo with a single thread and 100ms thinking time was set to be 0. AlphaZero(s=16k, t=800k) refers to 16000 search simulations after 800000 training steps.

Num. Sims	UCT const. (C)	Expl. (mvd)	Expl. (mvis)	Expl. (mval)
10	1.0	2168	2449	2173
10	2.0	2058	2408	2341
10	5.0	1902	2615	2517
10	10.0	1738	2555	2360
10	13.0	1799	2517	2598
10	20.0	1821	2830	2349
10	26.0	1888	2861	2669
100	1.0	1489	1509	1333
100	2.0	1404	1587	1395
100	5.0	1239	1145	1094
100	10.0	1213	1195	1245
100	13.0	1218	1292	1227
100	20.0	1350	1456	1342
100	26.0	1448	1747	1568
1000	1.0	1323	1218	1177
1000	2.0	1069	1212	864
1000	5.0	699	778	681
1000	10.0	697	601	632
1000	13.0	741	759	744
1000	20.0	859	962	991
1000	26.0	966	1029	1057
10000	1.0	1348	948	1134
10000	2.0	911	877	763
10000	5.0	516	582	538
10000	10.0	490	485	480
10000	13.0	511	465	470
10000	20.0	572	505	505
10000	26.0	631	575	570

Table S5: Average exploitability (in mbb/h) over five policy constructions obtained by independent searches of IS-MCTS runs at each information state in Leduc Poker. The parameter C is the value of the UCT exploration constant. The final policy is obtained either by normalizing the visit counts (mvd), choosing the action with maximum visits (mvis), or choosing the action with the maximal Monte Carlo value estimate (mval).

**Parameter**	**DQN**	**Tabular Q-Learning**	**A2C**
Learning rate (lr)	1e-1, 1e-2, 1e-3, 1e-4	NA	Actor lr: 1e-3, 1e-4, 1e-5<br>Critic lr: 1e-2, 1e-3
Decaying exploration rate	1., 0.8, 0.5, 0.2, 0.1	NA	NA
Replay buffer size	100, 1000, 10000, 100000	NA	NA
Hidden layer size	'32', '64', '128',<br>'32, 32', '64, 64'	NA<br>NA	'32', '64', '128',<br>'32, 32', '64, 64'
Num. of critic updates before every actor update	NA	NA	4, 8, 16
Step size	NA	0.1, 0.2, 0.5, 0.8, 1.0	

Table S6: Hyper parameters swept over in each RL algorithm.

REFERENCES

1. A. L. Samuel, Some studies in machine learning using the game of checkers. *IBM J. Res. Dev.* **44**, 206–226 (2000).
2. S. J. Russell, P. Norvig, *Artificial Intelligence: A Modern Approach* (Pearson Education, ed. 3, 2010).
3. D. Silver, A. Huang, C. J. Maddison, A. Guez, L. Sifre, G. van den Driessche, J. Schrittwieser, I. Antonoglou, V. Panneershelvam, M. Lanctot, S. Dieleman, D. Grewe, J. Nham, N. Kalchbrenner, I. Sutskever, T. Lillicrap, M. Leach, K. Kavukcuoglu, T. Graepel, D. Hassabis, Mastering the game of Go with deep neural networks and tree search. *Nature* **529**, 484–489 (2016).
4. M. Campbell, A. J. Hoane, F.-H. Hsu, Deep blue. *Artif. Intell.* **134**, 57–83 (2002).
5. G. Tesauro, TD-Gammon, a self-teaching backgammon program, achieves master-level play. *Neural Comput.* **6**, 215–219 (1994).
6. D. Silver, T. Hubert, J. Schrittwieser, I. Antonoglou, M. Lai, A. Guez, M. Lanctot, L. Sifre, D. Kumaran, T. Graepel, T. Lillicrap, K. Simonyan, D. Hassabis, A general reinforcement learning algorithm that masters chess, shogi, and Go through self-play. *Science* **362**, 1140–1144 (2018).
7. M. B. Johanson, "Robust strategies and counter-strategies: From superhuman to optimal play," thesis, University of Alberta, Edmonton, Alberta, Canada (2016).
8. M. Moravcik, M. Schmid, N. Burch, V. Lisý, D. Morrill, N. Bard, T. Davis, K. Waugh, M. Johanson, M. Bowling, Deepstack: Expert-level artificial intelligence in heads-up no-limit poker. *Science* **358**, 508–513 (2017).
9. N. Brown, T. Sandholm, Superhuman AI for heads-up no-limit poker: Libratus beats top professionals. *Science* **360**, 418–424 (2017).
10. N. Brown, T. Sandholm, Superhuman AI for multiplayer poker. *Science* **365**, 885–890 (2019).
11. N. Bard, J. N. Foerster, S. Chandar, N. Burch, M. Lanctot, H. F. Song, E. Parisotto, V. Dumoulin, S. Moitra, E. Hughes, I. Dunning, S. Mourad, H. Larochelle, M. G. Bellemare, M. Bowling, The Hanabi challenge: A new frontier for AI research. *Artif. Intell.* **280**, 103216 (2020).
12. A. Lerer, H. Hu, J. Foerster, N. Brown, Improving policies via search in cooperative partially observable games. *Proceedings of the Thirty-Fourth AAAI Conference on Artificial Intelligence* (AAAI, 2020).
13. J. Serrino, M. Kleiman-Weiner, D. C. Parkes, J. B. Tenenbaum, Finding friend and foe in multiagent games. *Proceedings of the Thirty-third Conference on Neural Information Processing Systems* (NeurIPS, 2019).

14. E. Lockhart, N. Burch, N. Bard, S. Borgeaud, T. Eccles, L. Smaira, R. Smith, Human-agent cooperation in bridge bidding. *Proceedings of the Cooperative AI Workshop at 34th Conference on Neural Information Processing Systems* (NeurIPS, 2020).
15. O. Vinyals, I. Babuschkin, W. M. Czarnecki, M. Mathieu, A. Dudzik, J. Chung, D. H. Choi, R. Powell, T. Ewalds, P. Georgiev, J. Oh, D. Horgan, M. Kroiss, I. Danihelka, A. Huang, L. Sifre, T. Cai, J. P. Agapiou, M. Jaderberg, A. S. Vezhnevets, R. Leblond, T. Pohlen, V. Dalibard, D. Budden, Y. Sulsky, J. Molloy, T. L. Paine, C. Gulcehre, Z. Wang, T. Pfaff, Y. Wu, R. Ring, D. Yogatama, D. Wünsch, K. McKinney, O. Smith, T. Schaul, T. Lillicrap, K. Kavukcuoglu, D. Hassabis, C. Apps, D. Silver, Grandmaster level in StarCraft II using multi-agent reinforcement learning. *Nature* **575**, 350–354 (2019).
16. T. W. Anthony, T. Eccles, A. Tacchetti, J. Kramár, I. M. Gemp, T. C. Hudson, N. Porcel, M. Lanctot, J. Pérolat, R. Everett, S. Singh, T. Graepel, Y. Bachrach, Learning to play no-press Diplomacy with best response policy iteration. *Thirty-third Conference on Neural Information Processing Systems* (NeurIPS, 2020).
17. J. Gray, A. Lerer, A. Bakhtin, N. Brown, Human-level performance in no-press Diplomacy via equilibrium search. *Proceedings of the International Conference on Learning Representations* (ICLR, 2020).
18. A. Bakhtin, D. Wu, A. Lerer, N. Brown, No-press Diplomacy from scratch. *Proceedings of the Thirty-fourth Conference on Neural Information Processing Systems* (NeurIPS, 2021).
19. N. Brown, T. Sandholm, Safe and nested subgame solving for imperfect-information games. *Proceedings of the 31st Conference on Neural Information Processing Systems* (NIPS, 2017).
20. M. Šustr, M. Schmid, M. Moravčík, N. Burch, M. Lanctot, M. Bowling, Sound search in imperfect information games. *Proceedings of the International Conference on Autonomous Agents and Multiagent Systems* (AAMAS, 2020).
21. V. Kovařík, M. Schmid, N. Burch, M. Bowling, V. Lisý, Rethinking formal models of partially observable multiagent decision making. *Artif. Intell.* **303**, 103645 (2022).
22. M. Schmid, "Search in imperfect information games," thesis, Charles University, Prague, Czech Republic (2021).
23. D. E. Knuth, R. W. Moore, An analysis of alpha-beta pruning. *Artif. Intell.* **6**, 293–326 (1975).
24. T. A. Marsland, M. Campbell, A survey of enhancements to the alpha-beta algorithm. *ACM Annual Conference* (Association for Computing Machinery, 1981), pp. 109–114.

25. J. Schaeffer, A. Plaat, CSC '96: New advances in alpha-beta searching. *ACM Conference on Computer Science* (1996), pp. 124–130.
26. S. Gelly, L. Kocsis, M. Schoenauer, M. Sebag, D. Silver, C. Szepesvári, O. Teytaud, The grand challenge of computer Go: Monte Carlo tree search and extensions. *Commun. ACM* **55**, 106–113 (2012).
27. L. Kocsis, C. Szepesvári, Bandit based Monte-Carlo planning, in *Machine Learning: ECML 2006. ECML 2006. Lecture Notes in Computer Science, Vol. 4212*. J. Fürnkranz, T. Scheffer, M. Spiliopoulou, Eds. (Springer, 2006), pp. 282–293.
28. R. Coulom, Efficient selectivity and backup operators in Monte-Carlo tree search, in *Computers and Games. CG 2006. Lecture Notes in Computer Science, Vol. 4630*. H. J. van den Herik, P. Ciancarini, H. H. L. M. J. Donkers, Eds. (Springer, 2007), pp. 72–83.
29. C. B. Browne, E. Powley, D. Whitehouse, S. M. Lucas, P. I. Cowling, P. Rohlfshagen, S. Tavener, D. Perez, S. Samothrakis, S. Colton, A survey of monte carlo tree search methods. *IEEE Trans. Comput. Intell. AI Games* **4**, 1–43 (2012).
30. D. Silver, J. Schrittwieser, K. Simonyan, I. Antonoglou, A. Huang, A. Guez, T. Hubert, L. Baker, M. Lai, A. Bolton, Y. Chen, T. Lillicrap, F. Hui, L. Sifre, G. van den Driessche, T. Graepel, D. Hassabis, Mastering the game of Go without human knowledge. *Nature* **550**, 354–359 (2017).
31. M. Zinkevich, M. Johanson, M. Bowling, C. Piccione, Regret minimization in games with incomplete information. *Advances in Neural Information Processing Systems 20* (NIPS, 2008), pp. 905–912.
32. S. Hart, A. Mas-Colell, A simple adaptive procedure leading to correlated equilibrium. *Econometrica* **68**, 1127–1150 (2000).
33. O. Tammelin, N. Burch, M. Johanson, M. Bowling, Solving heads-up limit Texas Hold'em. *Proceedings of the 24th International Joint Conference on Artificial Intelligence* (IJCAI, 2015).
34. M. Bowling, N. Burch, M. Johanson, O. Tammelin, Heads-up limit Hold'em poker is solved. *Science* **347**, 145–149 (2015).
35. M. Johanson, N. Bard, M. Lanctot, R. Gibson, M. Bowling, Efficient nash equilibrium approximation through Monte Carlo counterfactual regret minimization. *Proceedings of the Eleventh International Conference on Autonomous Agents and Multi-Agent Systems* (AAMAS, 2012).
36. N. Burch, M. Johanson, M. Bowling, Solving imperfect information games using decomposition. *Proceedings of the Twenty-Eighth AAAI Conference on Artificial Intelligence* (AAAI, 2014).

37. N. Brown, A. Bakhtin, A. Lerer, Q. Gong, Combining deep reinforcement learning and search for imperfect-information games. *Thirty-fourth Annual Conference on Neural Information Processing Systems* (NeurIPS, 2020).
38. R. Zarick, B. Pellegrino, N. Brown, C. Banister, Unlocking the potential of deep counterfactual value networks. arXiv:2007.10442 [cs.AI] (20 July 2020).
39. N. Brown, T. Sandholm, B. Amos, Combining deep reinforcement learning and search for imperfect-information games. *Proceedings of the Thirty-second Conference on Neural Information Processing Systems* (NeurIPS, 2018).
40. P. I. Cowling, E. J. Powley, D. Whitehouse, Information set Monte Carlo tree search. *IEEE Trans. Comput. Intell. AI Games* **4**, 120–143 (2012).
41. J. Long, N. R. Sturtevant, M. Buro, T. Furtak, Understanding the success of perfect information Monte Carlo sampling in game tree search. *Proceedings of the Twenty-Fourth AAAI Conference on Artificial Intelligence, AAAI'10* (AAAI, 2010), p. 134–140.
42. J. Nijssen, M. Winands, Monte Carlo tree search for the hide-and-seek game scotland yard. *IEEE Trans. Comput. Intell. AI Games* **4**, 282–294 (2012).
43. J. Nijssen, "Monte-Carlo tree search for multi-player games," thesis, Maastricht University, Maastricht, The Netherlands (2012).
44. J. Heinrich, D. Silver, Smooth UCT search in computer poker. *Proceedings of the 24th International Joint Conference on Artificial Intelligence* (IJCAI, 2015).
45. V. Lisý, M. Lanctot, M. Bowling, Online Monte Carlo counterfactual regret minimizationfor search in imperfect information games. *Proceedings of the Fourteenth International Conference on Autonomous Agents and Multi-Agent Systems* (AAMAS, 2015), pp. 27–36.
46. M. Lanctot, K. Waugh, M. Zinkevich, M. Bowling, Monte Carlo sampling for regret minimization in extensive games. *Advances in Neural Information Processing Systems 22* (NIPS, 2009), pp. 1078–1086.
47. J. Heinrich, M. Lanctot, D. Silver, Fictitious self-play in extensive-form games. *Proceedings of the 32nd International Conference on Machine Learning* (ICML, 2015).
48. M. Lanctot, V. Zambaldi, A. Gruslys, A. Lazaridou, K. Tuyls, J. Perolat, D. Silver, T. Graepel, A unified game-theoretic approach to multiagent reinforcement learning. *Advances in Neural Information Processing Systems* (NeurIPS, 2017).

49. H. Li, K. Hu, S. Zhang, Y. Qi, L. Song, Double neural counterfactual regret minimization. *Proceedings of the Eighth International Conference on Learning Representations* (ICLR, 2019).
50. N. Brown, A. Lerer, S. Gross, T. Sandholm, Deep counterfactual regret minimization. arXiv:1811.00164 [cs.AI] (1 November 2018).
51. E. Steinberger, A. Lerer, N. Brown, DREAM: Deep regret minimization with advantage baselines and model-free learning. arXiv:2006.10410 [cs.LG] (18 June 2020).
52. S. Srinivasan, M. Lanctot, V. Zambaldi, J. Pérolat, K. Tuyls, R. Munos, M. Bowling, Actor-critic policy optimization in partially observable multiagent environments. *Advances in Neural Information Processing Systems* (NeurIPS, 2018).
53. E. Lockhart, M. Lanctot, J. Pérolat, J.-B. Lespiau, D. Morrill, F. Timbers, K. Tuyls, Computing approximate equilibria in sequential adversarial games by exploitability descent. *Proceedings of the 28th International Joint Conference on Artificial Intelligence* (IJCAI, 2019).
54. D. Hennes, D. Morrill, S. Omidshafiei, R. Munos, J. Perolat, M. Lanctot, A. Gruslys, J.-B. Lespiau, P. Parmas, E. Duenez-Guzman, K. Tuyls, Neural replicator dynamics. *Proceedings of the International Conference on Autonomous Agents and Multiagent Systems* (AAMAS, 2020).
55. A. Gruslys, M. Lanctot, R. Munos, F. Timbers, M. Schmid, J. Perolat, D. Morrill, V. Zambaldi, J.-B. Lespiau, J. Schultz, M. G. Azar, M. Bowling, K. Tuyls, The advantage regret-matching actor-critic. arXiv:2008.12234 [cs.AI] (27 August 2020).
56. J. Perolat, R. Munos, J.-B. Lespiau, S. Omidshafiei, M. Rowland, P. Ortega, N. Burch, T. Anthony, D. Balduzzi, B. D. Vylder, G. Piliouras, M. Lanctot, K. Tuyls, From Poincaré recurrence to convergence in imperfect information games: Finding equilibrium via regularization. *Proceedings of the The Thirty-eighth International Conference on Machine Learning* (ICML, 2021).
57. S. McAleer, J. Lanier, P. Baldi, R. Fox, XDO: A double oracle algorithm for extensive-form games. *Proceedings of the Thirty-fifth Conference on Neural Information Processing Systems* (NeurIPS, 2021).
58. X. Feng, O. Slumbers, Z. Wan, B. Liu, S. M. McAleer, Y. Wen, J. Wang, Y. Yang, Neural auto-curricula in two-player zero-sum games. *Proceedings of the Thirty-fifth Conference on Neural Information Processing Systems* (NeurIPS, 2021).
59. J. Perolat, B. D. Vylder, D. Hennes, E. Tarassov, F. Strub, V. de Boer, P. Muller, J. T. Connor, N. Burch, T. Anthony, S. McAleer, R. Elie, S. H. Cen, Z. Wang, A. Gruslys, A. Malysheva, M. Khan, S. Ozair, F. Timbers, T. Pohlen, T. Eccles, M. Rowland, M. Lanctot, J.-B. Lespiau, B. Piot, S.

Omidshafiei, E. Lockhart, L. Sifre, N. Beauguerlange, R. Munos, D. Silver, S. Singh, D. Hassabis, K. Tuyls, Mastering the game of Stratego with model-free multiagent reinforcement learning. *Science* **378**, 990–996 (2022).

60. T. Dash, S. N. Dambekodi, P. N. Reddy, A. Abraham, Adversarial neural networks for playing hide-and-search board game Scotland Yard. *Neural Comput. Appl.* **32**, 3149–3164 (2020).
61. J. N. Foerster, F. Song, E. Hughes, N. Burch, I. Dunning, S. Whiteson, M. Botvinick, M. Bowling, Bayesian action decoder for deep multi-agent reinforcement learning. arXiv:1811.01458 [cs.MA] (4 November 2019).
62. S. Sokota, E. Lockhart, F. Timbers, E. Davoodi, R. D'Orazio, N. Burch, M. Schmid, M. Bowling, M. Lanctot, Solving common-payoff games with approximate policy iteration. *Proceedings of the Thirty-Fifth AAAI Conference on Artificial Intelligence* (AAAI, 2021).
63. D. Strouse, K. R. McKee, M. Botvinick, E. Hughes, R. Everett, Collaborating with humans without human data. *Proceedings of the Thirty-fifth Conference on Neural Information Processing Systems* (NeurIPS, 2021).
64. A. Bakhtin, N. Brown, E. Dinan, G. Farina, C. Flaherty, D. Fried, A. Goff, J. Gray, H. Hu, A. P. Jacob, M. Komeili, K. Konath, M. Kwon, A. Lerer, M. Lewis, A. H. Miller, S. Mitts, A. Renduchintala, S. Roller, D. Rowe, W. Shi, J. Spisak, A. Wei, D. Wu, H. Zhang, M. Zijlstra, Human-level play in the game of Diplomacy by combining language models with strategic reasoning. *Science* **378**, 1067–1074 (2022).
65. T. S. D. Team, Stockfish: Open source chess engine (2021); https://stockfishchess.org/.
66. M. Johanson, Measuring the size of large no-limit poker games. arXiv:1302.7008 [cs.GT] (27 February 2013).
67. Game of the year 1983: Scotland Yard; https://www.spiel-des-jahres.de/spiel-des-jahres-1983-scotland-yard/.
68. F. Southey, M. Bowling, B. Larson, C. Piccione, N. Burch, D. Billings, C. Rayner, Bayes' bluff: Opponent modelling in poker. *Proceedings of the Twenty-First Conference on Uncertainty in Artificial Intelligence* (UAI, 2005), pp. 550–558.
69. A. Elo, The Rating of Chessplayers, Past and Present (Arco Pub., ed. 2, 1986).
70. T. G. D. Team, Gnugo (2009); https://www.gnu.org/software/gnugo/.
71. P. Baudis, J. loup Gailly, Lemonsqueeze, Pachi: Software for the board game of go / weiqi / baduk (2016); https://pachi.or.cz/.

72. E. Jackson, Slumbot NL: Solving large games with counterfactual regret minimization using sampling and distributed processing. *Proceedings of the Computer Poker and Imperfect Information: Papers from the AAAI 2013 Workshop* (AAAI, 2013).
73. E. Jackson, Slumbot github repository; https://github.com/ericgjackson/slumbot2017.
74. N. Burch, M. Schmid, M. Moravčík,, M. Bowling, AIVAT: A new variance reduction technique for agent evaluation in imperfect information games. arXiv:1612.06915 [cs.AI] (20 December 2017).
75. V. Lisý, M. Bowling, Equilibrium approximation quality of current no-limit poker bots. *Workshops at the Thirty-First AAAI Conference on Artificial Intelligence* (AAAI, 2017).
76. R. B. Segal, On the scalability of parallel UCT. *CG'10: Proceedings of the 7th international Conference on Computers and Games* (Springer, 2010), pp. 36–47.
77. P. J. Huber, Robust estimation of a location parameter. *Ann. Math. Stat.* **35**, 73–101 (1964).
78. N. Brown, T. Sandholm, Strategy-based warm starting for regret minimization in games. *Proceedings of the Thirtieth AAAI Conference on Artificial Intelligence* (AAAI, 2016), pp. 432–438.
79. M. Lanctot, E. Lockhart, J.-B. Lespiau, V. Zambaldi, S. Upadhyay, J. Pérolat, S. Srinivasan, F. Timbers, K. Tuyls, S. Omidshafiei, D. Hennes, D. Morrill, P. Muller, T. Ewalds, R. Faulkner, J. Kramár, B. D. Vylder, B. Saeta, J. Bradbury, D. Ding, S. Borgeaud, M. Lai, J. Schrittwieser, T. Anthony, E. Hughes, I. Danihelka, J. Ryan-Davis, OpenSpiel: A framework for reinforcement learning in games. arXiv:1908.09453 [cs.LG] (26 August 2019).
80. M. Šustr, V. Kovařík, V. Lisý, Monte Carlo continual resolving for online strategy computation in imperfect information games. *Proceedings of the 18th International Conference on Autonomous Agents and Multiagent Systems* (AAMAS, 2019), pp. 224–232.
